# Responses to the Islamic headscarf in everyday interactions depend on sex and locale: A field experiment in the metros of Brussels, Paris, and Vienna on helping and involvement behaviors

**DOI:** 10.1371/journal.pone.0254927

**Published:** 2021-07-29

**Authors:** Martin Aranguren, Francesco Madrisotti, Eser Durmaz-Martins, Gernot Gerger, Lena Wittmann, Marc Méhu

**Affiliations:** 1 Centre National de la Recherche Scientifique, Unité de Recherches Migrations et Sociétés, Université de Paris, Paris, France; 2 Webster Vienna Private University Vienna, Vienna, Austria; Shahjalal University of Science and Technology, BANGLADESH

## Abstract

The Islamic headscarf has been in the middle of heated debates in European society, yet little is known about its influence on day-to-day interactions. The aim of this randomized field experiment (n = 840) is to explore how the generally negative views that surround the hijab in Europe manifest in the behavior that people direct to hijab-wearing women in everyday situations. Using a helping scenario and videotapes of the resulting interactions, we measured whether passengers offered assistance and also various details of behavior that indicate interpersonal involvement. We predicted that in interaction with the covered confederate less help would be offered, that women’s level of nonverbal involvement would increase but men’s decrease, and that responses would be stronger in Paris, intermediate in Brussels, and weaker in Vienna. We analyzed the data using Generalized Linear Models estimated with Bayesian inference. While the headscarf does not produce concluding differences in “overt” helping, it does affect “subtle” cues of interpersonal involvement. In response to the hijab, women across sites increase, but men in Paris decrease, the level of involvement that they show with their nonverbal behavior.

## Introduction

In Europe, probably no other piece of cloth has been the object of so much contradictory hermeneutic work in public debates as has the Islamic headscarf or *hijab*. While the available interpretations cover the entire spectrum from very negative (e.g. a symbol of women’s oppression [1]) to very positive (e.g. an expression of dignity and self-esteem [1]), in Europe the most common attitude towards the hijab consistently appears to be one of opposition, even among liberals [2–5]. At the same time, surveys also indicate some variation in the level of disapproval that the hijab encounters across national and institutional contexts in Europe. About 85% of French respondents support a ban on the headscarf in public places including schools, but the percentage is nearly 70% in Wallonia (the French-speaking region of Belgium), slightly more than 60% in Flanders (the Dutch-speaking region surrounding Brussels) and slightly less than 60% in Austria [6, 7].

The aim of this exploratory study is to examine how the negative views that surround the hijab in these European countries manifest in the behavior that people direct to hijab-wearing women in everyday situations. In stark contrast with the wealth of academic and nonacademic publications that have dealt with the hermeneutics of the Islamic headscarf, the differences in behavior that the hijab elicits in everyday interaction in European cities have received very limited attention on the part of researchers and the wider public. To fill this void, we conducted a field experiment on platforms of the metros of Brussels, Paris, and Vienna in which a confederate actress, following a helping scenario, initiated interactions with randomly selected passengers either wearing a hijab or with uncovered hair. The outcomes of interest are the probability with which passengers help the confederate and the level of interactional involvement that passengers show through nonverbal cues such as interpersonal distance, eye contact, or speech duration. Does passengers’ probability of helping and level of nonverbal involvement change when the headscarf is worn? Do women and men in Brussels, Paris, and Vienna respond in the same manner?

So far, we do not know if the differences in evaluation between social groups captured by surveys map into more or less subtle differences in behavior in everyday settings, or what specific differences in behavior such differences in evaluation bring about, resulting in a major research gap. These differences in behavior, as expressions of differences in social evaluations, could be acting as the “messages” whereby social recognition is recurrently given or withheld, taken or denied, and so disparities in social worth between social groups created and reproduced on a day-to-day basis. While social scientists have been analyzing educational or economic gaps for decades, the systematic study of such “recognition gaps” [8] is still at its beginning, warranting at the present stage, and as a step logically prior to theory testing, the conduct of exploratory work aimed primarily at “establishing the phenomena” [9]. We regard the present research as a contribution to this emerging field. Here we look at the “encoder” side of the process of communicating recognition in interpersonal relations, inquiring into the form of the behavioral changes that people show in interaction with a devalued other. We leave momentarily aside the “decoder” side of the process, that is the analysis of the specific meanings that the devalued other might attribute to those behavioral changes.

Convenience dictated the choice of Paris and Vienna as observation sites, as these are the cities where are based the two research teams involved in this project. In terms of exploration, the comparison was relevant because of the obvious differences in language and culture, in history of State-Church relations and in geographic origin of the populations associated with Islam (Maghreb, sub-Saharan Africa and Turkey in France but Turkey and ex-Yugoslavia in Austria). Brussels was added at a later stage as a (convenient) complement to the Parisian study, on the assumption that the linguistic, institutional and demographic contrast between Paris and Brussels was less marked than the one between these and Austria. Furthermore, in the years immediately preceding the experiments reported here, Paris and Brussels had been the stage of terrorist attacks perpetrated in the name of fundamentalist Islam, possibly exacerbating the responses to the hijab in these cities if their residents associated the garment to jihadi violence.

To our knowledge, this is the first cross-national field experiment that examines not only explicit helping behavior (which has received some attention in the literature [10–12]) but also the nonverbal involvement cues that dwellers of European cities show as they interact in a public place with a non-immigrant woman wearing the Islamic scarf. In this sense, we also contribute to research on nonverbal behavior by taking to the field the recording and measurement apparatus traditionally confined to the laboratory. By looking at the probability of providing assistance, we sought to add to the long-lasting literature on everyday discrimination based on the “helping behavior paradigm” [13]. The inclusion of nonverbal measures of involvement, in turn, was motivated by research underscoring the often “subtle” (vs. “overt” or “blatant”) expression of intergroup feelings in contemporary Western societies [14, 15]. We wished to explore if, as could be derived from this literature, “subtle” nonverbal cues expressed more faithfully participants’ true perceptions and evaluations than more “overt” helping behaviors. Assuming that people generally desire to appear unprejudiced to self and other, on the one hand, and that verbal offers of help are easier to monitor and control than nonverbal signs of involvement, on the other, we reasoned that nonverbal behavior might offer a more reliable window into people’s biased views.

As an experimental setting, metro platforms possess several desirable properties. They offer a highly predictable environment to which urbanites from very diverse backgrounds arrive in random order, where they stay for a few minutes only, and that they quit together and definitely as the train arrives. This facilitates randomization and replication, guards against interference between experimental units, and ensures wide demographic and socioeconomic coverage, enhancing internal and external validity.

### Overview of the procedure

In order to prepare the ground for deriving our predictions, we now offer a quick overview of the procedure, of which a detailed description is available in the Methods section.

On a platform of the local metro, a nonimmigrant confederate actress, using the same clothing across the three cities, approaches randomly selected passengers asking them for help. In one experimental condition, she appears with a hijab; in the other, with uncovered hair. The rest of the clothing is identical, as is the script she follows while interacting with the passengers. The script divides the interaction in two stages involving different verbal contents and body postures. The first stage consists in locating items on a portable map and leads confederate and passenger to position themselves side-by-side. In the second stage, the confederate shifts to a close face-to-face position, asking the passenger to estimate the duration of the trip ahead of her. After the passenger’s answer, the confederate laments being late for an important appointment, emphasizes that she needs to contact the person she has to meet, but regrets that her cell phone has run out of battery. After the passenger’s reply to this indirect request, a researcher intercedes to unmask the plot and to inform the passenger that the interaction has been videotaped, requesting consent to process the images. If time permits, the passenger is invited to answer to a short sociodemographic questionnaire.

The outcomes of interest are the passenger’s reply to the request for help and the nonverbal involvement behaviors that the passenger shows during the second stage of the interaction. This stage begins with the shift from a side-by-side to a face-to-face position and ends with the completion of the confederate’s indirect request, representing an observation period of 10 to 15 seconds. In this observation period, by changing her nonverbal behavior the confederate takes the initiative to increase the level of involvement of the ongoing exchange. The shift in position leads her to stand at a close distance with a direct body orientation toward the passenger. Moreover, she gazes at the passenger continuously during the entire observation period. Consequently, the passenger’s nonverbal behaviors of interest represent adjustments in response to the confederate’s initiative to intensify the level of interactional involvement. Do passengers compensate the confederate’s increase in involvement by decreasing theirs or do they reciprocate with further involvement on their part? Is compensation or reciprocation equally likely if the confederate wears or not the hijab?

### Conceptual framework

Our conceptual starting point is the hypothesis that, in the context of social interaction, the evaluation that interaction partner A makes of interaction partner B will influence the behaviors that A directs to B. If the outcome of a person’s evaluation is understood to operate on a continuum from very good to very bad, the hypothesis states more specifically that the degree of positivity-negativity of the evaluation that A makes of B will affect the behaviors that A directs to B.

Our empirical starting point is the above-cited fact that residents of Belgium, France, and Austria generally disapprove the practice of wearing the hijab in public. If disapproval of the hijab as a practice is allowed to imply a negative evaluation of the person who engages in the disapproved practice of hijab-wearing, the logical consequence is that women who wear the hijab in these countries will generally be the target of negative evaluations. Supporting this presumption, on the basis of a French-speaking Belgian sample a laboratory experiment concluded that participants were not well able to distinguish between dislike of Muslims as persons and dislike of allegedly Muslim ideas, values, and practices [16].

In the context of our experiment, the basic expectation is that, owing to the general tendency to disapprove the hijab in these three countries, passengers engaged in a social interaction with a woman of whom they have no personal knowledge will treat her differently if she wears the Islamic headscarf. Laboratory experiments based on student samples in the UK [17] and Germany [18] confirm that participants hold more negative views of, and respond more negatively to, women who wear the scarf, compared to women with uncovered hair.

To investigate the possible differences in treatment associated with the hijab, we consider two distinct types of behavior: helping and nonverbal involvement.

#### Helping

With regard to helping, we derive our predictions from the simple premise that the probability of offering assistance to the confederate is a linear function of the evaluation that passengers make of her. Assuming that a more negative evaluation leads to a lower probability of offering assistance, we predict that passengers will help the confederate less often when she wears the hijab. A field experiment conducted in train stations across three German provinces confirms this expectation, finding that an immigrant woman whose bag accidentally fell received less assistance from bystanders when she wore the headscarf [11]. Similarly, a Swiss field experiment performed in in the vicinity of Bern’s central train station found that pedestrians were less likely to help a young woman running late for a doctor’s appointment and needing to call ahead when she wore a hijab [12]. However, another Swiss field experiment carried out in public places in Zürich investigating the effects of the hijab in the context of a request for support to a political initiative did not establish a difference attributable to the Islamic scarf in the probability that pedestrians stop and listen or provide a supportive signature [10].

#### Nonverbal involvement

To formulate predictions about the involvement outcomes, we use the “sequential functional model of nonverbal exchange” [19]. This model was devised to account for some robust facts that its predecessor, the “arousal model of intimacy exchange” [20], failed to accommodate. Importantly for our experiment, these facts contradict the predictions that the arousal model makes about how an actor should respond to a change in nonverbal intimacy level initiated by an interaction partner. In these models, nonverbal intimacy is defined as the degree of union with, or openness toward, or liking of, or interest in, another person that nonverbal behavior is supposed to express or indicate.

The arousal model starts with the assumption that changes in intimacy levels in the course of an interaction precipitate arousal in the recipient, that the recipient rates that arousal somewhere on the valence continuum, and that the resulting evaluation determines whether the recipient’s nonverbal response is one of compensation or, alternatively, reciprocation. If the arousal precipitated by the other’s increase in intimacy is negatively valenced, the actor is expected to compensate with intimacy behaviors of opposite sign. If arousal is positively valenced, the expectation is that the actor will reciprocate or match the higher level of intimacy that the other proposes.

Now, a series of studies demonstrated, surprisingly but robustly, that when the encounter begins with a negative view of the interaction partner (or “alter), the actor (or “ego”) responds to increases in intimacy initiated by the negatively viewed partner not with compensation, as predicted, but in fact with reciprocation or matching [21–23]. In some of these studies, the level of reciprocation was found to be even higher with negatively than with positively valenced partners. This unexpected circumstance led to the interpretation that actors in an exchange with an unfavorably viewed interaction partner were exaggerating their level of intimacy, presumably to make the interaction more pleasant. The deliberate nature of the performance suggests that intimacy behaviors may be less spontaneous than initially thought.

To distinguish nonverbal behaviors from the intimacy function, the founding stone of the “sequential functional model of nonverbal exchange” is the more comprehensive notion of *nonverbal involvement behaviors* [19]. These designate behaviors that generally define the degree of cognitive engrossment or affective engagement manifested between participants in a social interaction. Interpersonal distance, eye contact, and speech duration, for example, are instances of behaviors that, at the level of the social interaction, usually function to manifest interpersonal involvement; closer distances, more intense eye contact (especially in the role of the listener [24]), and longer speech duration represent changes in nonverbal behavior that increase involvement. The model posits that, at the level of the individual interactant, involvement behaviors can serve distinct functions, of which two are directly relevant here, namely expressing intimacy (the only one that the previous model contemplated) and exercising “social control”, broadly understood as a sub-type of purposive or instrumental behavior.

Greater liking for, or interest in, another person generally results in increased intimacy with that person. Now, according to the model not all intimacy manifests in nonverbal involvement and, conversely, nonverbal involvement may serve functions other than expressing intimacy. For our purposes, the most important alternative function is using involvement behaviors not to give outer expression to an inner feeling of union, openness, liking, or interest but to exercise “social control.” Involvement behaviors acquire a “social control” function when they are performed with the deliberate intention of influencing the behavior of others. Behaviors that signal increased involvement such as closeness, forward lean, or an animated tone of voice might be recruited in direct efforts to persuade others or less directly in strategies of “impression management” [25] designed, for example, to show interest and awaken a favorable disposition in the other interactant.

The sequential functional model [19] makes a contrast between involvement behaviors that serve the “social control” function, which are characterized as more self-conscious and managed, and involvement behaviors that serve the intimacy function, which are depicted as more spontaneous and affective. Thus, involvement behaviors such as distance, eye contact, and speech duration might function to express intimacy or alternatively to manage impressions.

But what function will prevail? A field experiment conducted in Washington D.C. predicted interpersonal behavior in public with hijab-wearing women, as here, from negative views of the hijab in the society at large [26]. In line with the “intimacy expression” hypothesis, the study revealed that female confederates who entered shops asking for job openings were the target of subtle forms of negativity when they wore the hijab, compared to a control condition in which they appeared with uncovered hair. The measure of subtle negativity, under the heading “interpersonal discrimination” [27], covered among others ratings of involvement behaviors such as interpersonal distance, affirmative gestures, smiling, and eye contact. Additionally, and analysis on measures of the length of conversations indicated that interaction partners spoke less with the hijab-wearing confederate. In Europe, however, to our knowledge no field experiments have examined the effects of the Islamic scarf on involvement behaviors. Although not concerned with the hijab, a field experiment in the Paris metro examined if the stigma widely attached to the Roma in Europe transpires in differences in involvement behaviors when passengers interact with a woman wearing a glaringly Romani skirt [28]. In line with the “impression management” hypothesis, female passengers (but not male passengers) turned out to interact at closer distances when the confederate was recognizable as a Roma person.

#### Differences between the sexes

The sex of the passenger might moderate the size or sign of the effect of the hijab on helping and involvement behavior. There are at least three candidate reasons for this. A first possible source of different responses among men and women is the fact that men, including Muslim men in Islamic States where the hijab is commonplace, consistently judge women who wear the hijab less facially attractive than women who appear with uncovered hair [29, 30]. If attractiveness has an impact on overall person evaluation, and assuming that women’s judgments of attractiveness are unaffected by the hijab, men’s appraisals of hijab-wearing women should be more negative than those of women, leading to a more important decrease in nonverbal involvement and similarly in the probability of offering assistance (“attractiveness hypothesis”).

A second possible reason for sex differences in the pattern of response to the hijab is that women might be more inclined than men to see the hijab-wearing woman as a victim of male oppression [31, 32], perhaps as a result of a greater concern with care compared to men [33]. Consequently, women should increase involvement and help more often to express positive intimacy and offer adequate care to a person considered to be in need of protection (“victimization hypothesis”).

A third possible reason is that in Belgium, France, and Austria, women appear to be more “agreeable” than men [34–36], a personality trait exemplified by adjectives such as sympathetic, kind, warm, helpful, or friendly. To that extent, the motivation to make the exchange more pleasant can be expected to be more frequent among women. If making the interaction more pleasant is the goal of nonverbal involvement reciprocation when the other is negatively viewed, it follows that reciprocation in the hijab condition should be more frequent among women than among men (“agreeableness hypothesis”).

Although the posited mechanisms differ, these three distinct hypotheses converge in the prediction that women should reciprocate involvement more than men, and conversely that men should compensate (i.e. decrease involvement) more than women, when the confederate wears the hijab. The attractiveness and victimization hypotheses, in addition, predict that female passengers will also provide more assistance to the veiled confederate.

#### Differences between cities

The city where the interaction takes place can be expected to give rise to differences in the effect of the hijab both on the probability of offering assistance and the level of nonverbal involvement that passengers show. The above-cited evidence indicates that negativity toward the Islamic headscarf is higher in France, intermediate in Belgium, and lower in Austria. We expect the differences in behavior associated with the hijab to follow this order. In line with this, if people tend to associate the hijab with terrorist attacks perpetrated in the name of Islam, we expect differences in behavior to be more extreme in Paris and Brussels (where such attacks had occurred before the experiment) than in Vienna (where none had taken place at that point).

Tu sum up, here is a schematic list of the predictions that we set out to explore:

*Prediction 1*: *Help less likely*. In response to the confederate’s indirect request for the passenger’s mobile phone, passengers will less often help the confederate when she wears the hijab.

*Prediction 2*: *Involvement*. 2a. In response to the confederate’s increase in nonverbal involvement (close distance, direct body orientation, gaze directed to the passenger’s eyes), passengers will increase involvement when she wears the hijab (follows from the hypothesis of involvement as deliberate impression management).

2b. In response to the confederate’s increase in nonverbal involvement, passengers will decrease involvement when she wears the hijab (follows from the hypothesis of involvement as spontaneous expression of intimacy).

*Predictions 3–5*: *Sex moderates the effect of the hijab*. 3. The effects expected by Prediction 1 will be stronger for male than for female passengers.

4. The effects expected by Prediction 2b will be stronger for male than for female passengers.

5. The effects expected by Prediction 2a will be stronger for female than for male passengers.

*Predictions 6–8*: *Locale moderates the effect of the hijab*. The effects expected by Predictions 1 and 2a/2b will be

6. Stronger in Paris than in Brussels,

7. Stronger in Paris than in Vienna, and

8. Stronger in Brussels than in Vienna.

## Method

### Design

This field experiment follows a between-subjects design in which roughly equal numbers of male and female passengers in Brussels, Paris, and Vienna were randomly assigned to interacting with a confederate who appeared either with uncovered hair or wearing an Islamic headscarf. The assignment and subsequent interaction took place within metro stations that are themselves embedded within cities. The most important criteria for selecting the set of sampled stations were suitability for the envisaged procedure and representativeness of the city’s diversity. As explained above, we expect a significant part of the variability external to our manipulation to be attributable to the sex of the passenger and the city where the encounter takes place. Hence, the most relevant “blocks” or clusters in this experiment are subgroups of passengers defined by their sex and the city where they interacted with the confederate. As the city is entirely confounded with the person of the confederate, we will use the more neutral term “site” to refer to unique combinations of a city and a confederate. The aim of this design is to put us in a position to assess the “simple effects” of the hijab within each of the six groups emerging from the combination of the two levels of sex and the three levels of site, and to assess averages of these simple effects within levels of one factor and across levels of the other (for example, the average of simple effects among women across the three sites, or the average of simple effects among women and men in Brussels). Based on previous studies [28] and on pilot work indicating that this sample size could be sufficient to detect small to moderate effects, we aimed to recruit at least 120 passengers within each site-sex group, equally distributed across experimental conditions and sampled stations. The interactions between the confederate and the passengers were recorded on video and audio and the resulting identifying data were processed only with the permission of the passenger.

### Stations selection

Within each city, passengers interacted with the confederate on the platform of a metro station. While we aimed equal numbers of stations within each city, the method of selection could not be exactly the same at each site.

In chronological order, the first city where the data was collected was Paris. In that city, stations were selected at random using a set of filters. The first filter consisted in eliminating all the stations in the upper and lower quartiles by number of passengers, which was a convenient way of taking into account the fact that packed and deserted stations would not offer a suitable environment for the experiment. With the stations in the mid quartiles a random list was then created. The second filter involved visiting the stations in the order stipulated by the random list and ascertaining that the platform was assigned to a single direction (not two) and physically arranged in such a way that there was a single entrance (not many) placed on one of the two longitudinal extremes (not in the middle) of the platform. Compared to other arrangements, platforms with these physical features maximize assay productivity by excluding two sources of uncertainty. Having a single entrance means that, during field sessions, the research team knows exactly where the next passenger will be coming from. The fact that the platform serves a single direction, combined with the fact that platforms are equipped with time displays, means that the team has a generally good estimation of the time at which all passengers present at a given point on the platform will leave. Thus the six stations retained for the Paris study result from the random selection of stations with similarly arranged platforms that receive intermediate numbers of passengers going all in the same direction. These are: Boucicaut, Michel Bizot, Notre Dame de Lorette, Pyrénées, Ranelagh, and Riquet.

In Vienna, stations were selected with several criteria in mind: station configuration, frequentation rates, and socio-economic status. In order to keep as much similarity with the selection of the Paris stations, we selected stations in which each platform serves a single direction, the main difference with Parisian stations being that most (if not all) platforms in Vienna have two, sometimes three, entrances. Regarding frequentation rates, we avoided stations served by multiple metro lines, as these have the highest rates. Based on passenger counts provided by the *Wiener Linien* (Viennese subway operator) collected between September and December 2017 only stations with mid tertile passenger frequencies were preselected to avoid over- or under-populated stations. This was confirmed by separate passenger frequency counts conducted by the researchers on separate days within the intended testing interval of 10 am to 2 pm. The last criterion was to select a set of stations that would cover a range of socio-economic conditions. Although Vienna presents a rather balanced socio-economic landscape (with neither extremely poor, nor extremely rich areas) and little geographical segregation due to religious belonging, we selected two stations from lower, medium, and higher socio-economic areas, respectively. This was also to address the observation that, in Vienna, poorer areas tend to have a higher proportion of individuals whose main religion is Islam. The six stations selected for the Vienna are Herrengasse, Schweglerstrasse, Taborstrasse, Troststrasse, Unter St Veit, and Zieglergasse.

In Brussels, a number of pilot visits to randomly selected stations made it clear that, in contrast to Paris, most stations gave the impression of being deserted. Hence, we tried to identify the stations where the numbers of passengers that populated the platforms were roughly comparable to those of the stations that we had sampled in the French capital. Only stations that serve as hubs, and therefore the most populated stations of the Brussels metro network, appeared to satisfy this demand. Lacking a sufficient number of eligible stations in terms of density to allow ourselves to be selective, we did not attempt to ensure similarity regarding the physical arrangement of the platforms. However, while we started off with six eligible stations, we eventually eliminated one (station Beekant) precisely because the physical arrangement of the platform created complications for our procedure (more precisely, the platform served two directions). So the five selected stations in Brussels are relatively dense ones within the local metro network, and their physical arrangement, while not formally identical, is with regard to our experiment functionally similar to that of the Parisian stations. The selected Bruxellois stations are Arts-Loi, Gare Centrale, Gare du Nord, Louise, and Rogier.

### Sampling

After a pilot study in March 2018, data collection in Paris proceeded between May and June of the same year. Acting as an institutional review board, the CNRS correspondent of the French commission for the protection of privacy and confidentiality CNIL approved the study and the transportation authority RATP gave us formal clearance to conduct the experiment in the metro. We made five data collection visits within each of the six selected stations. Every visit had a duration of two hours. To neutralize potentially systematic effects of the weekday and the hour at which assays were performed, we took the following measures. Visits to the same station were scheduled on different weekdays. Of the two hours in which each visit consisted, we assigned the first hour to one experimental condition and the second hour to the other condition, balancing for the entire experiment, both within and across stations, the number of times that each condition was placed first or second in chronological order.

During the hour devoted to each condition, we sought to recruit an equal number of male and female passengers, which involved the combination of a method for approximating random selection and another one for stratifying the sampling of men and women. Random selection was approximated with a method of systematic selection: during the time period comprised between the departure of the last train and the arrival of the following one, the confederate approached the first passenger who arrived at the platform. The stratification technique consisted in starting with the method of systematic selection regardless of the sex of the passenger, recruiting one passenger (say, a man), and then reapplying the method of systematic selection but only to passengers of the opposite sex (women). The third passenger was again selected regardless of sex, the fourth by stratifying by sex, and so on. This means that, in stratifying our sample, we relied on our own commonsensical understandings of sexual dimorphism to identify passengers as men or women, and not on passengers’ self-reported gender identity. Data collection visits took place on regular weekdays between 12pm and 2pm. In Paris, this is the only period of the working day in which waiting times are in the range of 3–5 minutes (instead of 1–2), maximizing the chances that the confederate will get to complete the script before the following train arrives.

In Vienna, data collection took place in November and December 2018, and March 2019, to accommodate the availability of the actress. Data were collected during week days, between 10am and 3pm, i.e. outside peak hours. It took a total of twenty-six days to complete testing in Vienna. The participants sampling technique was the same as in Paris. The data collection procedure received ethical approval from the institutional review board of Webster University and the *Wiener Linien* (the main transportation company in Vienna) granted clearance to perform the study on the metro premises.

In Brussels, after a series of short pilot visits in 2018, data collection took place between February and March 2019. Acting as an institutional review board, the CNRS correspondent of the French commission for the protection of privacy and confidentiality CNIL approved the study and the transportation authority STIB gave us formal clearance to conduct the experiment in the metro. Except for the morning and evening rush hours, the stations we selected were suitable for sampling at any time during the rest of the working day. We took advantage of this by scheduling not one but two daily visits to two different stations. To minimize the undesirable effects of excessive fatigue, each of the visits had a duration of 90 minutes (and not 120 as in Paris). Using the same procedure as before, we allocated 45 minutes to each condition. The two successive daily visits took place between 11am-12:30pm and 1:30pm-3pm, respectively. The method of systematic stratified sampling was the same as in Paris.

### Procedure

#### a. Field team

**Fig 1:**
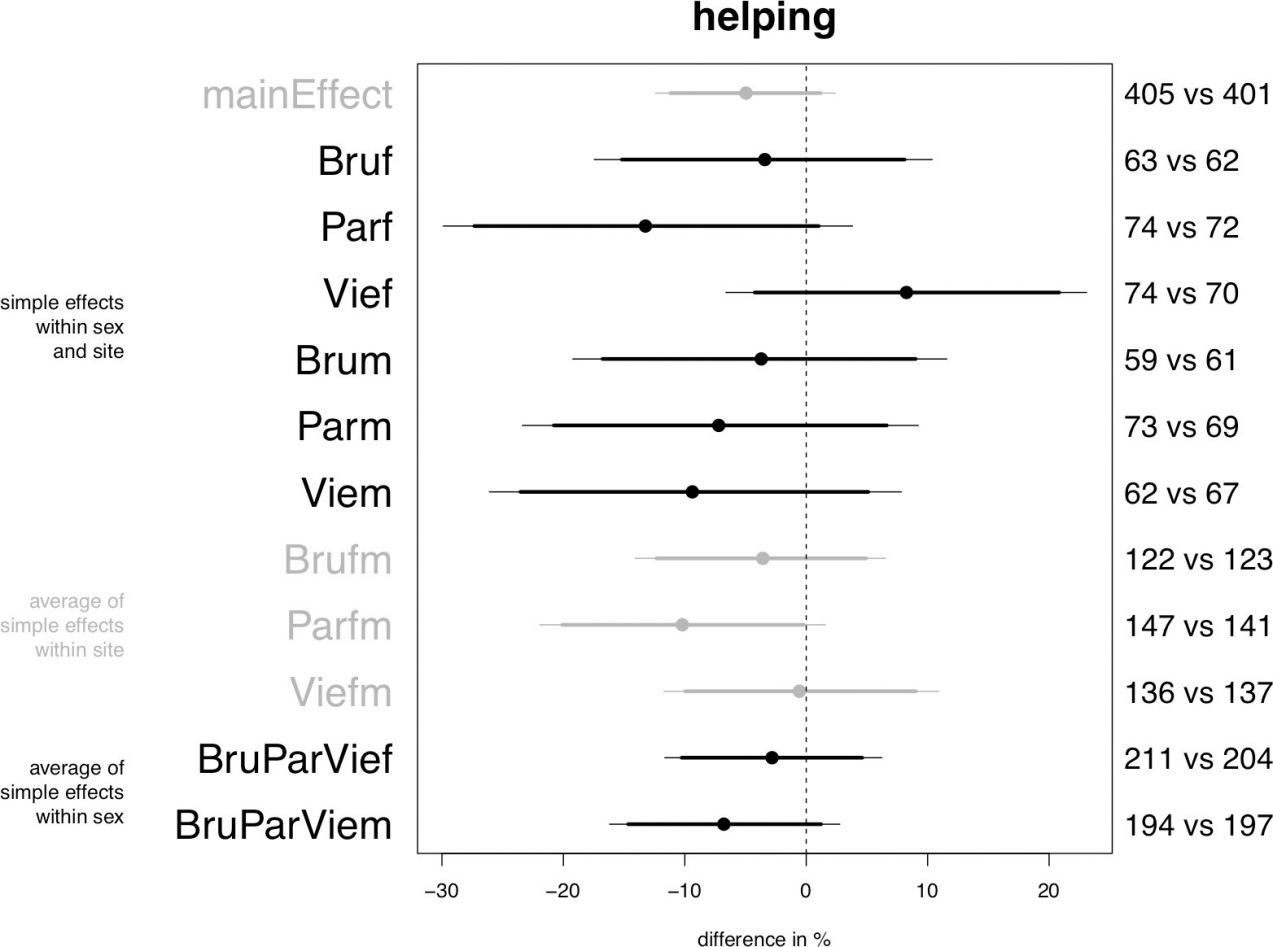
Planned model on helping, central posterior intervals. From left to right, the first column gives the heading of different groups of parameters. The second column specifies the individual parameters, where “Bru” stands for “Brussels”, “Par” for “Paris”, “Vie” for “Vienna, “f” for “female passengers” and “m” for “male passengers”. The quantities on the right-hand side of the plot specify the number of observations on which the estimation of the corresponding parameter directly relies; the first number refers to the sample size of the hijab group, the second number to that of the control group. The x-axis represents the difference between the control and the hijab conditions. Within the plot area, the dashed vertical line in the middle indicates the location of the value 0, which signifies no difference between the control and the hijab conditions. The horizontal segments represent the central 95% posterior intervals of the parameters. The bolder section of the segment corresponds to the central 90% posterior interval and the solid point indicates the median of the distribution. Our decision rule is to reject the null hypothesis of no effect of the hijab if the 95% or 90% posterior interval of a given parameter excludes the value zero. In graphical terms this implies that the thin (95%, alpha = 0.05) or bold (90%, alpha = 0.10) segment representing the parameter does not intersect the dashed vertical line. The differences in color are only meant to facilitate reading.

In the three cities, each data collection session was carried out by a team involving two experimenters and a professional actress performing the covered or uncovered confederate. In each city, the female confederate was a different person. For this role, we recruited women in their thirties who speak the local tongue as a first language, do not look like the stereotypical immigrant (by our commonsensical standards), but are nonetheless credible in their role of ordinary hijab-wearing women (we eventually confirmed their credibility in informal talks with Muslim participants). The confederate used the same type of clothing across cities, and at times the self-same piece of garment (see S1 File, Fig 1. The individual who appears in that image, namely the confederate from the Paris study, has given written informed consent (as outlined in PLOS consent form) to publish these case details). While the clothing match is nearly total, the match on height is far from perfect, as the Paris actress, compared to the local average, is taller, the Brussels actress slightly shorter, and the Vienna actress substantially shorter. The Brussels actress is from France, meaning that her French is native but her accent is not exactly local (*Bruxellois)*. In Vienna, the actress is from Germany (Bavaria) and does not have a local Viennese accent. As for the experimenters, Martin Aranguren and Francesco Madrisotti collected the data in Paris, Gernot Gerger and Lena Wittmann in Vienna, and Francesco Madrisotti and Eser Durmaz-Martins in Brussels.

#### b. Confederate’s script

The confederate carries a discreetly mounted portable microphone and an audio device to record the conversation with the passenger. She waits until the selected passenger stops walking and stays standing somewhere on the platform. The passenger stands typically in a position that is perpendicular, on the frontal or coronal plane, to the rails. The confederate, carrying a portable metro map, approaches walking parallel to the rails and stops when the tip of her shoe is at a rough 10 cm distance from the passenger’s. The result is a side-by-side arrangement in which confederate and participant form an approximate right angle on the frontal plane. Shortly before making the last step, the confederate begins the following (stylized) script:

*Confederate: Hello. Excuse me, I’m a bit lost. Could you please tell me where is, on this map, the station where we currently are?*

*Passenger: …*

*C: Thank you. And I’d like to go to station X (X is always on the same line, a few stations after the station where the interaction takes place)*

*P: …*

When the passenger finishes giving directions, the confederate shifts from the side-by-side to a face-to-face position using a fixed choreography of steps. If the passenger has not moved while giving the directions side-by-side, the newly created distance between the tips of the passenger’s and the confederate’s shoes will be roughly 25 cm (75 cm from heel to heel). As the confederate initiates the shift, she carries on as follows, looking in the eyes at the passenger until the end of the script:

*C: And how long do you think the trip will be?*

*P: …*

*C: Oh no! I’m late for an important appointment with a person. I absolutely need to tell the person that I’m late but unfortunately the batteries of my mobile are dead*.

*P: …*

The script is considered complete when the passenger does something that is interpretable as the reply to the confederate’s last line, consisting most of the times in an explicit refusal or acceptance to help the confederate in some way. If the passenger offers assistance, the confederate herself discloses the plot, while an experimenter approaches to inform the passenger about the project. If the passenger does not offer assistance, the confederate says:

*C: Still, thank you very much. I’ll make it in some way. Goodbye*.

In Paris and Brussels, the confederate then leaves the scene and the experimenter approaches the passenger directly. In Vienna, only the confederate discloses the plot, regardless of the passenger’s reply.

#### c. Experimenters’ script

**Fig 2:**
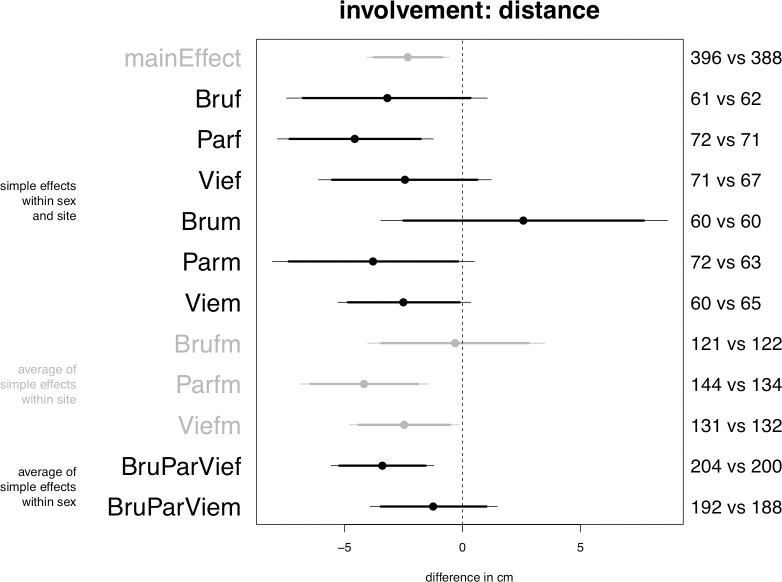
Planned model on distance, central posterior intervals.

Experimenter 1 is in charge of selecting the passenger, using the aforementioned technique of systematic stratified sampling, and discreetely videotaping the interaction with the confederate. Experimenter 1 carries an inconspicuous chest-mounted portable camera that he or she operates from a portable tablet (in Paris and Brussels, see S1 File, Fig 2) or mobile phone (in Vienna). To make the videos, Experimenter 1 places herself or himself at a few meters distance from the passenger on the same line, parallel to the rails, that the confederate follows to approach the passenger. That is, during the side-by-side stage of the interaction, Experimenter 1 videotapes the confederate’s back and the passenger’s profile, both full body. When the confederate shifts to the face-to-face position, the camera captures the profiles of both interactants.

Experimenter 2 is in charge of debriefing the passenger and requesting his or her informed consent to process the collected identifying data. To request informed consent, Experimenter 2 discreetely approaches the passenger while the interaction with the confederate is still underway. Once the script is complete, passengers are handed a leaflet with a short description of the project and contact information. Consent is given orally and videotaped in Paris and Brussels, whereas it is expressed in written form in Vienna. Experimenter 2 invites passengers who give consent to answer to a short orally- administered questionnaire on socio-demographic variables, registering the responses on a portable tablet. The procedure was approved by the CIL division of CNRS (Paris and Brussels experiments) and by the IRB of Webster University (Vienna experiment).

As she reaches the end of the script, the confederate does not directly ask participants if she can use their phone. Rather, she makes her request indirectly by stating that her phone is out of battery. Our previous experience with a similar procedure [28], as well as preparatory assays in the framework of the present research, indicated that the proportion of passengers who, after refusing to help the confederate, also refused to give consent to the researcher was lower when the confederate’s request was expressed in an indirect manner.

### Measurements and outcome variables

The demographic variables that were measured with the questionnaire are age, educational achievement, income, and religion.

The outcome variables analyzed in this article pertain only to the face-to-face part of the interaction between confederate and passenger. Unless otherwise specified, all the variables listed below are limited to this observation period. In particular for distance and eye contact, the observation period starts as the confederate begins the shift in position and ends with the end of the confederate’s last line (distance and eye contact enter a different, less readily interpretable regime at that point that would require separate analysis). Except for interpersonal distance, all outcome variables were coded using the software ELAN [37]. We agreed on a common set of coding rules for the Paris and Vienna teams but intercoder reliability assessments took place only within teams. To assess the reliability of the coding, for each variable a portion of the measurements were performed by two coders independently and their level of agreement quantified using Krippendorff’s alpha [38], which turned out to be above 0.7 in all the cases. In most measurements, coders were neither blind to the experimental condition nor unaware of the research objectives.

#### Speaking time of confederate and passenger

Every annotation begins with any vocalization, not only articulated speech, on the part of confederate or passenger, and ends when the vocalization ends. Vocalizations may include laughter, hesitations, and fragmented speech, for example. Speech pauses shorter than 500ms were not counted as pauses and were included in the continuous annotations. The outcome variable speech rate is the proportion of the observation period that the passenger spent vocalizing.

#### Number of steps

The technique used for coding interpersonal distance led us to measure, as a prior necessary step, the number of steps that the passenger made in response to the confederate’s shift from the side-by-side to the face-to-face position. A step occurs when some part of one of the passenger’s soles gets lifted from the ground, unless the final position of the sole is identical to the initial one. The observation window goes from 5 seconds prior to the beginning of the confederate’s shift in position to the end of the confederate’s last line in the script. Each step is coded with an annotation that begins with the step and ends with the beginning of the following step (or the end of the observation period). The outcome variable number of steps is simply the count of the step annotations. We also characterized steps as effecting a movement toward or away from the confederate. As the characterization is not error-free, and the overwhelming majority of the coded steps turn out to be away from the confederate, we eventually retained the step count as a reasonably good proxy of steps away.

#### Interpersonal distance

This outcome was defined as the maximal distance between the confederate’s farthest heel, relative to the passenger, and the passenger’s farthest heel, relative to the confederate. Operationally, we located the chronologically last annotation of a step, and we used the video frame located in the middle of this annotation to take the measurement. The measuring technique, relying on a screen ruler, consisted in counting the number of pixels comprised between the farthest heels of confederate and passenger, and then counting the number of pixels represented by the length of the confederate’s shoe (in Paris and Vienna) or the length of a tile (in Brussels). The outcome variable interpersonal distance is the ratio of the first measure and the second one, multiplied by the actual length in centimeters of the confederate’s shoe or the tile.

#### Eye contact

Annotations begin when the passenger starts to look at the confederate in the face and end when he or she stops doing so. If the quality of the image was insufficient to understand where exactly the passenger’s eyes were aiming, the orientation of the head was used instead. Eye contact was operationalized as two distinct outcome variables: i) the gaze rate while speaking, which is the total proportion of time that the passenger spent looking at the confederate while the passenger was speaking, and ii) the gaze rate while listening, which is the total proportion of time that the passenger spent looking at the confederate while the confederate was speaking. Only the gaze rate while listening was used in statistical analyses to avoid overlap with the speech rate, and therefore the possibility that the effect of the hijab on one of these outcomes may be mediated by the other. Further, the gaze rate while listening, given that looking at the speaker is a conventional display of attention or “advertence”, is a better indicator of involvement than the gaze rate while speaking or an overall gaze rate averaging over conversational roles [24].

#### Helping with the phone

For each complete interaction, we coded helping as a dichotomy: either the passenger cooperated or not after the confederate finished the last line of the script. The category cooperated-yes includes a variety of possible helping behaviors on the part of the passenger, such as inviting the confederate to dictate an SMS, asking the confederate to spell the phone number of the person she had to meet, or simply handing the mobile to her.

### Sample description

Table 1 shows, for each experimental condition within each site and sex group, the number of complete interactions and, from these, the number of times that the passenger gave consent to have his or her identifying data processed in the framework of the project. For each passenger who granted consent, we have at least one observation on at least one outcome variable. The total sample size is 840.

**Table 1:** Sample information by site-sex group.

	completed script		gave consent	
	control	hijab	control	hijab
Brussels, female	75	70	67	66
Brussels, male	75	63	65	58
Paris, f	83	81	78	76
Paris, m	80	82	71	74
Vienna, f	90	93	74	79
Vienna, m	79	70	68	64

For a variety of reasons, we were not able to measure all covariates and outcomes for all passengers, and consequently for a given site-sex group *n* changes according to the variable under examination. When it comes to the covariates (i.e. age, education, income) the most important of these reasons is the conjunction of a short span of time before the arrival of the train and a hurried passenger. Technical problems, especially low audio or image quality, account for most of the missing values on the outcome variables.

In terms of age, education, income, and religion, some very obvious differences between sites deserve mention. Except for age, the other sociodemographic characteristics were measured drastically less often in Brussels than in Paris and Vienna. The level of educational achievement is lower in Vienna, where the median is “upper secondary”, than in Brussels and Paris. Further, Paris is the site with the largest proportion of passengers in the highest and lowest (ca. 30%) monthly income strata (respectively, ca. 20% above 3,000 EUR and ca. 30% below 800 EUR). The proportion of passengers without a religion is highest in Paris (nearly 60%) and lowest in Vienna (below 40%), where in turn the proportion of Christians (around 50%) is largest. In the three cities, the proportion of Muslim male passengers in the sample is at least twice as large as the proportion of Muslim female passengers.

### Statistical analyses

Totaling 840 passengers, the sample on which the statistical analyses are based approximates a balanced distribution of participants between the experimental conditions, the sexes and the stations embededded within the cities where data collection was performed. Subsequently, the collected video and audio files were used to measure the outcome variables (see Method for more details).

We estimated four planned model analogous in logic to traditional ANOVAs but estimated with Bayesian inference in the context of the Generalized Linear Model [39].

Bayesian models have the important advantage of giving the analyst flexibility in the choice of the probability distribution to be used to describe the data. This leeway allowed us to model our continuous but bounded gaze and speech proportions not with the usual normal distribution, which is unbounded and symmetric, but with a more adequate beta distribution, which operates in the (0,1) interval and accommodates a wide range of distributional shapes that better describe the variability at work in our data. Further, to avoid the nuisances of outliers we have modeled our distance measurements using the *t*-distribution instead of the normal distribution. We have also taken advantage of this flexibility to estimate separate parameters for each site and sex group when it comes to variance, the “normality” parameter of the t-distributed distance observations, and the slopes associated of control predictors.

The common predictors in these models are the experimental condition, the sex of the passenger, the site where the confederate encountered the passenger, the two-way interactions between these factors and the three-way interaction. An additional predictor acting as a covariate is age, which was discretized into three groups of equal frequency within each site-sex cluster. The other sociodemographic covaratiates, namely education and income, were excluded due to an important proportion of missing values.

The outcome of the first model is the probability of helping the confederate. This dichotomous outcome was given a Bernoulli distribution. The outcome of the second model is distance as measured in cm. The distance measurements were given a t distribution to make the estimations robust to outliers [39]. The outcome of the third model is eye contact as measured by the proportion of time that the passenger spent looking at the confederate when the passenger was in the role of the listener. Similarly, the outcome of the fourth model is speech duration as measured by the proportion of time that the passenger took to speak to the confederate during the face-to-face part of the exchange. These proportion measurements, being continuous but bounded between 0 and 1, were given a Beta distribution [40]. The models on the Beta-distributed eye contact and speech duration measurements include distance as a control predictor, to take into account the possibility that changes in eye contact or speech duration might be functioning as adjustments to prior changes in interpersonal distance. In these models, the slope of the predictor distance is allowed to vary across groups defined by combinations of site and sex. Except for the Bernoulli-distributed model (which has no variance parameter), in the other models the variance of each site and sex cluster is not assumed to be homogeneous but estimated from the data. In the model on distance measurements, the parameter describing the “normality” of the t-distribution is also estimated from the data, with a separate parameter for each site and sex group.

The four planned Anova-like models obey the following linear function:

1. y.hat_[i]_ = b0 + b.ageGroup[sex_[i]_, site_[i]_] + b.cond[cond_[i]_] + b.sex[sex_[i]_] + b.site[site_[i]_] + b.cond.sex[cond_[i]_, sex_[i]_] + b.cond.site[cond_[i]_, site_[i]_] + b.sex.site[sex_[i]_, site_[i]_] + b.cond.sex.site[cond_[i]_, sex_[i]_, site_[i]_]

On the left of the formula, letter i indexes the n^th^ observation; y.hat_[i]_ is the value that the model predicts for the nth observation (to be distinguished from the value that the nth observation actually takes). We omit the link function to avoid cluttering, but it may be recalled that it is the identity function for the t-distributed distance measurements and the logit function for the Bernoulli-distributed helping outcomes and the Beta-distributed gaze and speech proportions. The addition on the right of the formula lists the predictors, where “cond” abbreviates “experimental condition”.

The Anova-like models on the gaze and speech proportions where estimated with and without distance as a control predictor. When the latter was included, its slope was allowed to vary by site and sex group. The corresponding linear function takes on the following form:

2 y.hat_[i]_ = b0 + b.ageGroup[sex_[i]_, site_[i]_] + b.cond[cond_[i]_] + b.sex[sex_[i]_] + b.site[site_[i]_] + b.cond.sex[cond_[i]_, sex_[i]_] + b.cond.site[cond_[i]_, site_[i]_] + b.sex.site[sex_[i]_, site_[i]_] + b.cond.sex.site[cond_[i]_, sex_[i]_, site_[i]_] + b.distance[sex_[i]_, site_[i]_]

The post hoc model estimating the effect of the hijab on the probability of not moving uses formula (1) and the logit link function. The post hoc model assessing the effect of the hijab on distance when failure to move is controlled obeys the following formula:

3 y.hat_[i]_ = b0 + b.ageGroup[sex_[i]_, site_[i]_] + b.cond[cond_[i]_] + b.sex[sex_[i]_] + b.site[site_[i]_] + b.cond.sex[cond_[i]_, sex_[i]_] + b.cond.site[cond_[i]_, site_[i]_] + b.sex.site[sex_[i]_, site_[i]_] + b.cond.sex.site[cond_[i]_, sex_[i]_, site_[i]_] + b.noSteps[noSteps_[i]_, sex_[i]_, site_[i]_],
  where “noSteps” indexes whether the passenger made at least one step during the observation period. Each site and sex group receives a separate slope estimating the effect of the variable noSteps on interpersonal distance. The post hoc model that evaluates the effect of the hijab on helping when the involvement behaviors are controlled is formulated as follows:
4 y.hat_[i]_ = b0 + b.ageGroup[sex_[i]_, site_[i]_] + b.cond[cond_[i]_] + b.sex[sex_[i]_] + b.site[site_[i]_] + b.cond.sex[cond_[i]_, sex_[i]_] + b.cond.site[cond_[i]_, site_[i]_] + b.sex.site[sex_[i]_, site_[i]_] + b.cond.sex.site[cond_[i]_, sex_[i]_, site_[i]_] + b.distance[sex_[i]_, site_[i]_] * distance_[i]_ + b.gazeRate[sex_[i]_, site_[i]_] * gazeRate_[i]_ + b.speechRate[sex_[i]_, site_[i]_] * speechRate_[i]_

#### Priors

Noninformative priors were set following [39]. In the Bernoulli- and Beta-distributed models, which operate on the logit scale, hyperparameters were given a normal distribution with a mean of 0 and a fixed variance of 2. In the t-distributed models, hyperparameters were given a normal distribution with a mean of 0 (with the exception of the hyperparameter corresponding to the intercept, which was given a mean equal to the data’s average) and fixed variance equal to the data’s standard deviation times 10.

#### MCMC sampling and checks

To approximate the posterior distribution of the parameters of interest we used Markov chain Monte Carlo (MCMC) sampling as implemented by the software Jags [41] via the programming language R [42]. Two important desiderata regarding the MCMC process are to achieve samples that are representative of the posterior distribution and that are of sufficient size to yield accurate and stable estimates. We checked that the samples were representative of the posterior distribution through visual examination of trace plots and density plots, on the one hand, and consideration of the Gelman-Rubin statistic of convergence, on the other. None of these checks gave any signs of unrepresentativeness. On the other hand, we checked that the generated samples were large enough (and therefore accurate and stable) by considering a measure called the “effective sample size”. The estimates of all the parameters reported below rest on effective sample sizes of at least 10,000, as recommended by [39].

The supplemental materials include the R scripts and the data frames used for estimating each model and drawing the plots, as well as tables presenting in numeric form the estimated central posterior intervals pertaining to each model.

*Note on reporting style*. When using Bayesian inference, results come in the form of probability distributions. Every parameter derived from the model (e.g. the simple effect of the experimental condition among women in Brussels) receives an individual posterior probability distribution, that is a list of all possible parameter values and their corresponding estimated probabilities, which together sum to 1. In a Bayesian framework, testing a null hypothesis amounts to asking if the posterior probability of the relevant parameter is sufficiently different from the parameter value 0. To facilitate this assessment, we offer a graphical display of the posterior 90% (alpha = 0.10) and 95% (alpha = 0.05) intervals representing the credible values of the parameters of interest. Being central, these intervals provide two-tailed hypothesis tests. The parameters represented in the plots quantify the main effect of the experimental condition, the simple effects within site and sex clusters, and averages of simple effects between levels of factor site within each level of factor sex (average effect among women or among men across sites), or between levels of factor sex within each level of factor site (average effect in Brussels, in Paris, or in Vienna across sex groups). At a given alpha level, when the posterior interval intersects the value 0 on the x axis of the plots, we accept the null hypothesis of no effect of the hijab. When the posterior interval does not intersect 0 on the x axis, we reject the null hypothesis at the corresponding alpha level.

## Results

### Helping

The model weakly confirms that passengers help less often the veiled confederate (Prediction 1) but that the decrease in helping is moderated by locale, with Paris standing out as the only site where this negative effect is in place (Prediction 4.1). The credible decrease in probability in Paris lies between slightly more than zero and 20% (but alpha = 0.10, see Fig 1, parameter “Parfm”).

### Distance

The model confirms Prediction 2a: passengers increase involvement with the covered confederate by standing at closer distances, as indicated by a credible main effect in the range from 1 to 4 cm (see Fig 2, “mainEffect”). Prediction 4 is also confirmed, as the overall effect among women turns out to be stronger than the overall effect among men. Thus, averaging over sites women increase involvement by reducing distance in the hijab condition by 1 to 6 cm (“BruParVief”), whereas the effect is not credible for the average of men (“BruParViem”). Supporting Predictions 6 and 7, the model shows that the effect is stronger or more likely in Paris (“Parfm”) than in Brussels (“Brufm”) and Vienna (“Viefm”). However, contradicting Prediction 8, the effect is less credible in Brussels than in Vienna. Of note, only in Paris is the increase among women strong enough to emerge as a credible simple effect at alpha = .05, estimated to be in the range from 1 to 8 cm.

### Eye contact

**Fig 3:**
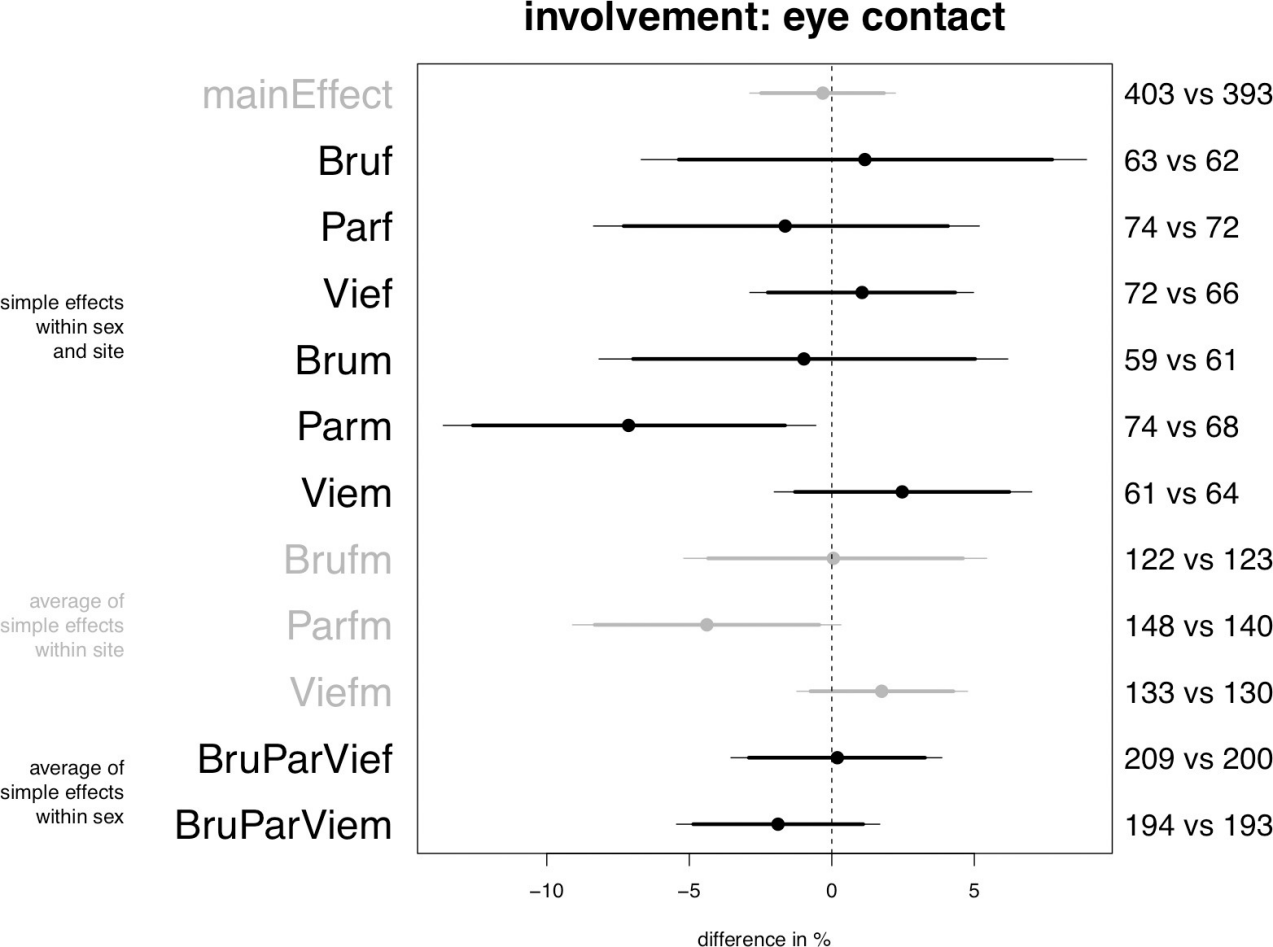
Planned model on eye contact, central posterior intervals.

The model supports the expectation that passengers will decrease involvement with the covered confederate by lowering the rate of eye contact (Prediction 2b), but the data indicates that this negative effect is moderated both by locale and by sex, in a manner which is consistent with our predictions. First, averaging over sex groups only in Paris is a 0.3% to 9% decrease in eye contact credible as an effect of the hijab (alpha = 0.10, see Fig 3, “Parfm”), as expected by Predictions 6 and 7. However, consistent with Prediction 5, when men and women are considered separately in Paris, it turns out that the negative effect is credible among men (“Parm”) but not among women (“Parf”). In Paris men decrease involvement in interaction with the covered confederate with a decrease in eye contact estimated to lie between 1% and 14% (parameter “Parm”). The results are qualitatively the same including or excluding distance as a control predictor.

### Speech duration

**Fig 4:**
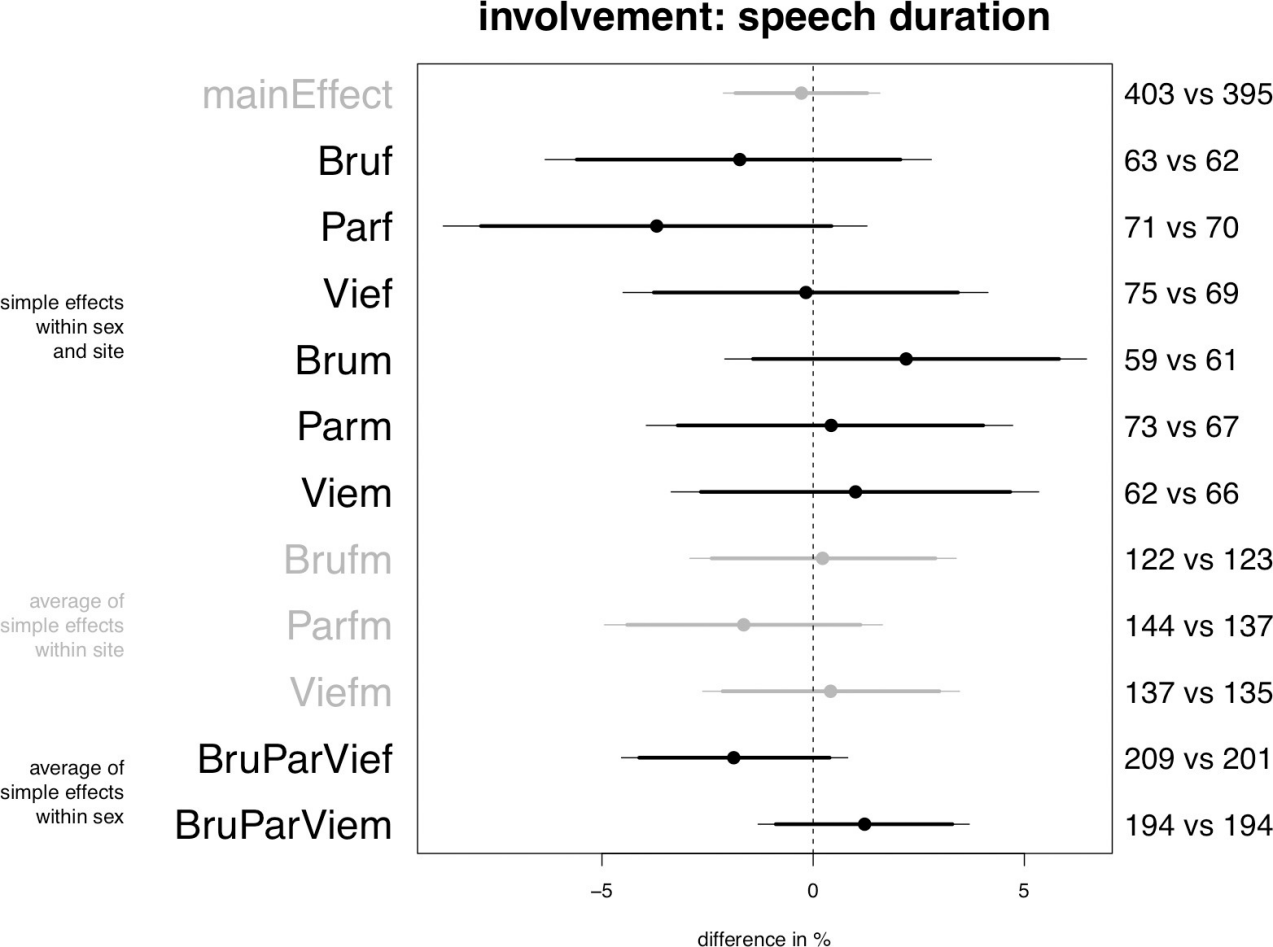
Planned model on speech duration, central posterior intervals.

The model on the speech duration measurements does not support any of the predictions (see Fig 4). The results are the same including or excluding distance as a control predictor.

### Post hoc models

After seeing the data, we performed a series of additional models to assist the interpretation of the results. The first two post hoc models assess if the decrease in distance associated with the hijab is not mediated by passenger’s failure to move. We reasoned that if passengers show closer distances because they fail to move, their immobility may be signalling something other than the acceptance or reciprocation of involvement that we had so far presupposed.

The first model of this mediation analysis is identical to the one performed on helping behavior, except that the outcome variable is failure to move, coded dichotomically as absence or presence of steps on the part of the passenger during the observation period.

**Fig 5:**
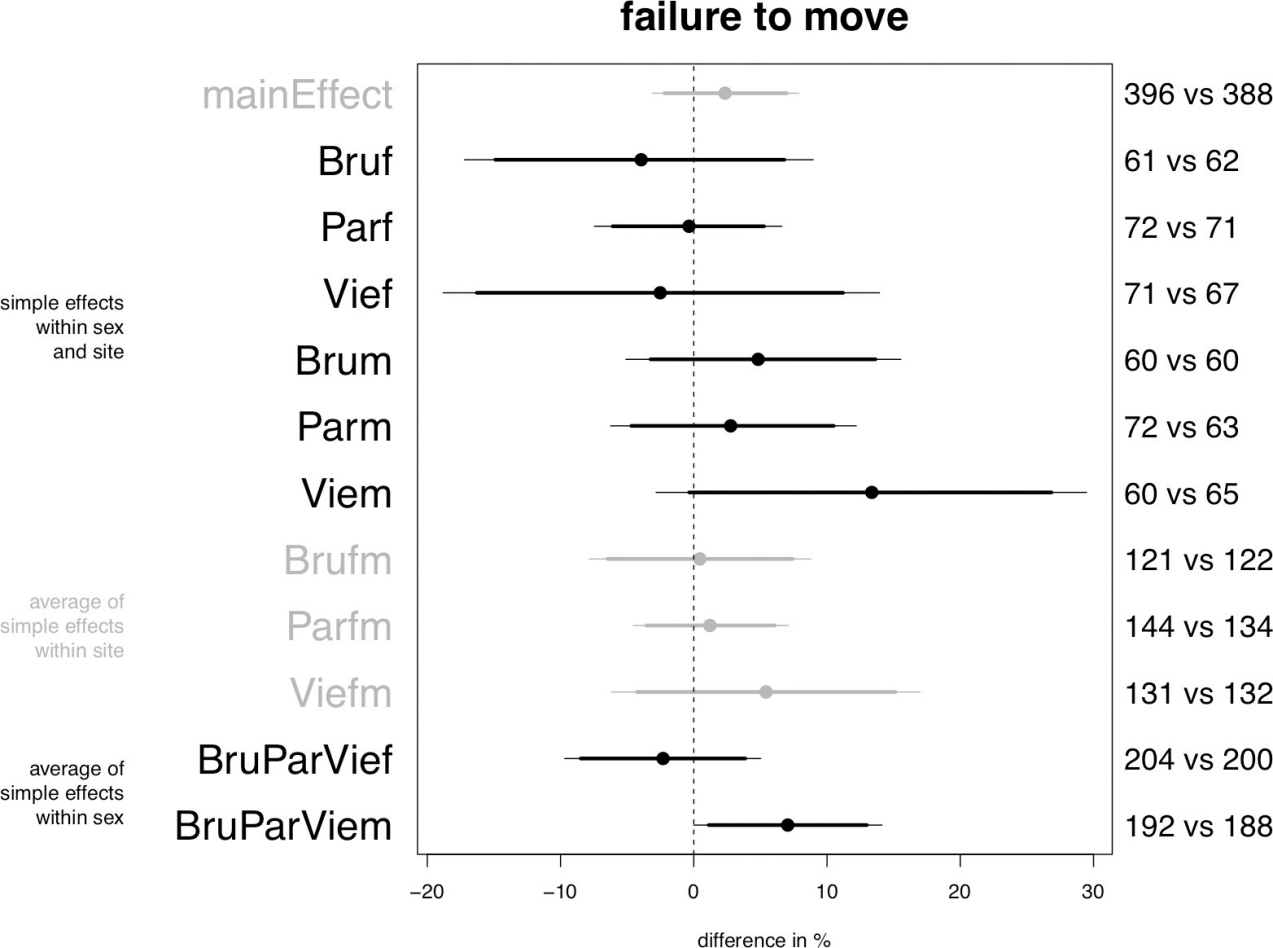
Post hoc model on the probability that the passenger fails to move.

The output presented in Fig 5 indicates that averaging over sites the hijab increases the probability that men fail to move by between slightly more than 0% and 14% (parameter “BruParViem”). The most impressive effect takes place among men in Vienna, where the central posterior interval of the corresponding parameter is close to being credible at alpha = 0.90 (“Viem”).

**Fig 6:**
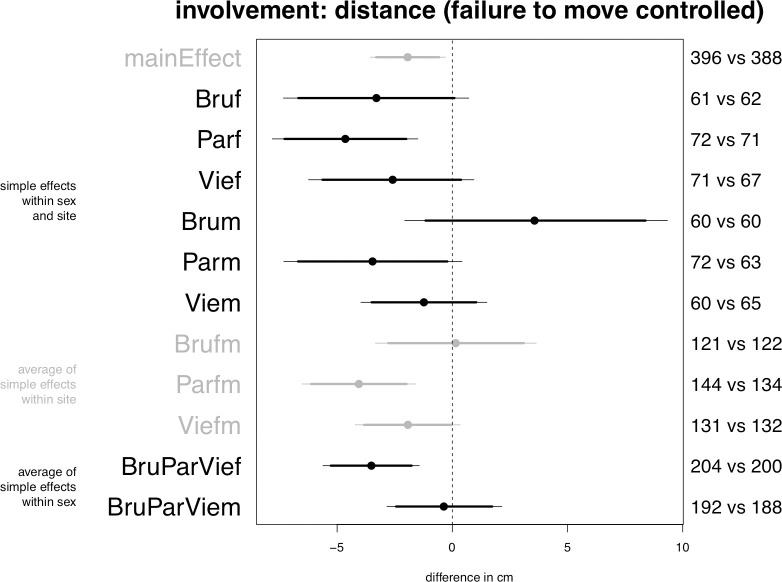
Post hoc model on distance with failure to move included as a control predictor.

The second model is identical to the one performed on distance, with the difference that this time failure to move is included as a control predictor whose slope is allowed to vary by sex and site combinations. This model’s output (see Fig 6) confirms that among men in Vienna failure to move partially mediates the decrease in interpersonal distance elicited by the hijab. Following the inclusion of failure to move as a predictor, the credibility of the effect among men in Vienna (“Viem”), and with it the credibility of the effect in Vienna averaging over men and women (“Viefm”), decreases, bringing the alpha level above 0.10 whereas in the planned model the effect was credible at alpha = 0.05. In the other groups the effects of the hijab on distance remain virtually identical to the ones estimated by the planned model.

**Fig 7:**
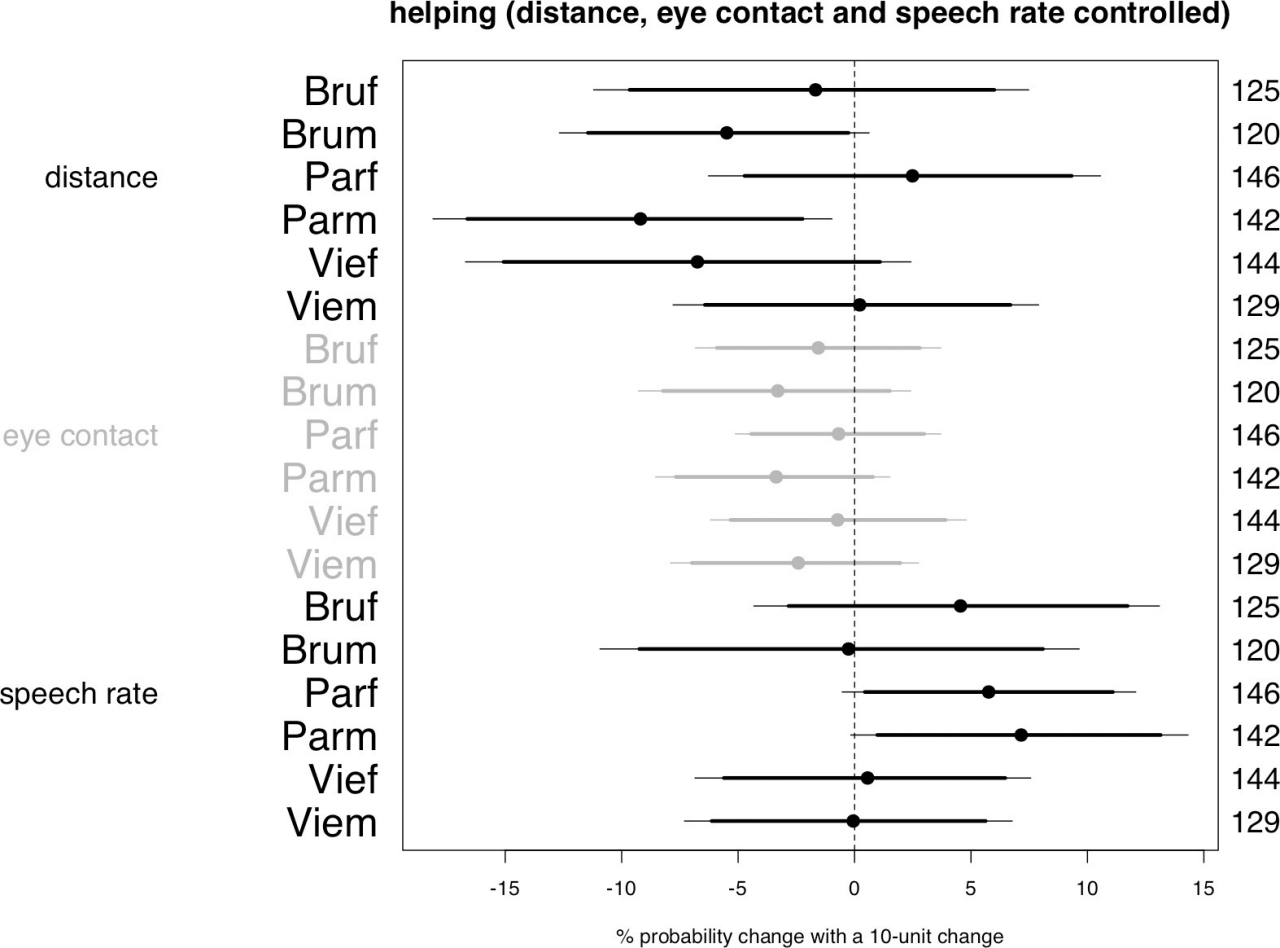
Post hoc model on helping with distance, eye contact, and speech rate controlled. The plot reports the central posterior intervals of the parameters estimating the slopes of the involvement outcomes (distance, eye contact, speech duration), treated as predictors of the probability of helping. The segments estimate the change in the probability of helping that is associated with a 10-unit change in distance (cm), gaze rate or speech rate (percentual points).

The third post hoc model assesses if the probability of helping is predicted by the involvement behaviors that the passenger displays before the request for help is uttered. This model is identical to the one performed above on the probability of helping, with the difference that distance, gaze rate, and speech rate are included as additional predictors, with varying slopes for each site and sex group. The output reprised in Fig 7 indicates that among men in Paris a 10 cm reduction in distance is associated with an increase in the probability of helping the confederate estimated to lie between 1% and 15% (distance, “Parm”). Men in Brussels exhibit the same effect but in weaker form (distance, “Brum”). In a similar vein, in Paris among women and men alike a 10% increase in speech rate is associated with an increase in the probability of helping the confederate ranging from slightly more than zero to 14% (alpha = 0.10, speech rate, “Parf” and “Parm”).

The last group of post hoc models tackles the following question: do the reported effects depend on whether the passenger perceives the confederate as a fellow member of his or her own group? The hypothesis of ingroup favoritism [43], and more generally the idea of homophily, leads to the prediction that the passenger’s group identity should moderate the effect of the hijab on his or her behavior. Operationally, we tested this prediction by estimating simple effects of the hijab among Muslims and non-Muslims, and also, within each category, among men and women. On this basis, we then explored interaction effects by inspecting the differences between simple effects.

Self-reported Muslims represent about 10% of the sample, totaling only 70 participants across cities. It is possible to seek to estimate the effect of the hijab among Muslims vs. non-Muslims at the level of the entire sample, and even within each sex group, but the number of Muslim participants is too small within sex and site sub-groups (e.g. women in Brussels, men in Vienna) to allow an assessment of differences in the effect of the hijab at this level. Hence, in this analysis we leave aside Predictions 6–8, that is those concerned with the effects of the site. When it comes to the remaining Predictions 1–5, the introduction of the principle of ingroup favoritism forces a contrast in sign (or direction) in their formulation. Thus, Predictions 1–5 hold intact for non-Muslims but are reversed in sign for Muslims. For example, in light of the hypothesis of ingroup favoritism we still expect passengers to help less often the veiled confederate, but only when they are non-Muslim. If they are Muslim, in contrast, we expect them to help *more* the confederate in the hijab condition, and the same logic applies to the other predictions.

The models used to this purpose are identical in all respects to the planned ones, except that in this post hoc analysis they include additional parameters to estimate the main effect of being Muslim, the two-way interactions between being Muslim and the experimental condition, on the one hand, and between being Muslim and the passenger’s sex, on the other, and the three-way interaction between being Muslim, the experimental condition and the passenger’s sex.

None of the models dealing with the probability of helping the confederate, gaze rate or speech rate support any of the predictions derived from the ingroup favoritism hypothesis, replicating within the groups delineated by the Muslim-yes vs. Muslim-no dichotomy the (nil) results previously reached with the planned models as regards the main effect of the hijab and its simple effect within sex groups across sites. Importantly, the model on the interpersonal distance measurements confirms Predictions 2a, and 4 for the sub-sample of non Muslim participants, yielding credible negative simple effects of the hijab both among non-Muslims in general (in the inteval [-4 cm, -1 cm]) and among non-Muslim women in particular ([-6 cm, -2 cm]). That is, we corroborate that the results arising from the planned model on distance capture effects of the hijab that concern mainly, if not exclusively, passengers who perceive the veiled confederate as an outgroup. In other words, the higher level of interpersonal involvement that passengers across sites, and women primarily, show by standing closer in interaction with the hijab-wearing confederate can be interpreted as an intergroup phenomenon.

An important caveat is in order, though. The proportion of participants who completed the questionnaire (reaching the question on religion, placed at the end of it) is 97% in Paris, 79% in Vienna but only 21% in Brussels. Consequently, the differences between Muslims and non Muslims averaging across sites that we have just reported represent with greater confidence the true tendencies at work in Paris and Vienna than those from Brussels.

## Discussion

We now offer a brief summary of the results of this experiment. There is weak evidence (at alpha = 0.10) that passengers offer assistance less often to the veiled confederate (Prediction 1), but this negative effect is confined to Paris (Predictions 6 and 7). By interacting at closer distances, passengers show greater involvement when the confederate wears the hijab (Prediction 2a), but the effect is moderated both by sex and locale. First, the decrease in distance is stronger among women than among men (Prediction 4). Second, the effect is stronger in Paris than in Brussels and Vienna (Predictions 6 and 7), but unexpectedly the effect is weaker in Brussels than in Vienna (contradicting Prediction 8). When it comes to eye contact, passengers do decrease involvement (Prediction 2b) but again the effect is limited to Paris (Predictions 6 and 7), and stronger among Parisian men than among Parisian women (Prediction 5). Our post hoc analyses indicate that the decrease in distance among men in Vienna is mediated by passenger’s failure to move, suggesting that within this group staying closer to the covered confederate might not be indicating greater involvement (this possibility is elaborated below). Bolstering the involvement hypothesis however, another post hoc model shows that among men in Paris shorter distances predict a higher probability of offering assistance to the confederate. Another post hoc analysis suggests that the higher levels of interpersonal involvement manifested in closer distances, especially among women, concerns primarily non-Muslim passengers, excluding the possibility of an artefactual finding that the operation of ingroup favoritism could explain away.

### Assessing the number and size of effects

In the abstract, the number of observed effects might strike the reader as being remarkably small. Yet, the opposite impression could arise from assessing the results in light of some details of the procedure. The behaviors we have measured take place in a time window comprised between 10 and 15 seconds. The difference between the control and the hijab condition is strictly limited to the presence or absence of the headscarf, with the rest of the clothing being identical, not ethnically connoted, and equally credible for covered and uncovered women of the confederate’s age. The confederate’s appearance makes her credible as a hijab-wearing woman but she does not connote by her accent or manners an immigrant or socially disadvantaged condition. In other words, the observation period and the intensity of the stimulus are minimal. With this in mind, one could be surprised that in spite of an observation window of minimal duration, a stimulus of minimal intensity, and an amount of random variability inevitably greater than in the laboratory, the procedure has been effective in uncovering any effects at all.

The size of the reported results on the involvement outcomes might also strike the reader as inconsequential. What difference can it make to interact closer in the order of 1 to 10 cm, or to decrease eye contact by in the order of 1 to 10%? To invite a fair appreciation of the size of the reported effects, we address two separate points, namely the relationship between evaluation and behavior and the distinction between the “encoding” and the “decoding” ends within the process of communicating social recognition.

At the time of launching the experiment, we ignored the type of function that would best describe the relationship between, on the one hand, differences in evaluation between covered and uncovered women (which the available evidence indicated was negative) and, on the other hand, differences in the behavior that people direct to covered and uncovered women. If the function is assumed to be linear, small differences in evaluation should map to small differences in behavior. Alternatively, if the function is assumed to be constant below a critical point, after which it is assumed to become linear, differences in evaluation do not map to differences in behavior unless they exceed that threshold of positivity or negativity. We know that on average the views of the hijab that residents of Belgium, France, and Austria hold are negative, but we ignore how negative they are on a continuous scale from very bad to very good. Is the negative reaction to the hijab of the same order as, for example, the negative reaction to terrorist murders committed in the name of Islam or of the same order as, for example, the negative reaction to the Islamic prohibition of consuming alcohol or pork? If the level of negativity associated with the hijab is near the very-bad pole, our results are disappointing, because extreme differences in evaluation would have been expected to give rise to more pronounced differences in behavior. But if the underlying level of negativity is intermediate to weak, then our results are effectively reflecting the behavioral manifestation of modest but true differences in social evaluation. If we drop the presumption that hijab-wearing women are in the same class as terrorist attacks (but why assume this?), and accept the alternative view that they are in the same class as other distinctive everyday religious practices of European Muslims, the expectation of large effects loses traction.

Aside from the problem of the relationship between evaluation and behavior, the effects reported might appear to be too weak to be perceived by the target, implying that they have no communicative import and are therefore of no consequence in the creation and reproduction of “recognition gaps” [8] between social groups. This is actually an empirical question that we have started to address in follow-up experiments. These unambiguously indicate that the target does perceive the difference in eye contact reported in the present article [44]. Similarly, the possibility that the credible difference found in interpersonal distance might be perceived by the interactional partner should not be dismissed out of hand, as we argue below.

In the lower half of the posterior interval quantifying the effect of the hijab among men in Paris, a 7% to 15% decrease in eye contact cannot occur without the intervention of some gross, potentially noticeable behaviors. More specifically, to decrease the rate of eye contact the most common intervening behavior is looking away, a behavior that typically involves not only decentering the eyes but also the head from the interaction partner’s face. When, as in this experiment, looking away breaks mutual gaze, it is not unreasonable to expect the interaction partner to notice the discontinuation, especially when the person initiating the break is in the role of the listener (because the norm of “advertence” requires the listener to gaze at the speaker [24]). A follow-up experiment confirms this suspicion [44]. The result here reported that men look less at the interaction partner when the latter is a hijab-wearing woman was replicated with a procedure involving an ultimatum game, again on platforms of the Paris metro, in which passenger and confederate interacted as equals. In a subsequent experiment in the same setting and involving again an ultimatum game, the treatment consisted in having a male confederate replay the pattern of eye contact found among the men who had encountered the hijab-wearing confederate in the previous experiments, including the one reported above. The male confederate interacted with a sample of randomly selected female passengers, with the aim of examining the average effect of this pattern of look (the “hijab-gaze”) among women. The experiment reveals that women who are the target of the “hijab-gaze” experience more negative affect and perceive that the interlocutor pays less attention. In other words, it is stressful to be the recipient of this look.

On the other hand, a difference in the range of 2 to 6 cm in interpersonal distance as that found among women across the three sites might seem insignificant at first sight. But at least three circumstances can invite to revise this assessment. First, one of the most robust results of decades of studies on interpersonal distance is that dyads composed of one female and one male interact at larger distances than dyads composed of two females [45, 46]. Looking at our data, the estimated difference in distance when the confederate interacts with a male as opposed to a female passenger (i.e. the main effect of sex) is in the range of 3 to 8 cm, an interval clearly comparable to the one estimated for the effect of the hijab among women. In other words, the main effect of sex on interpersonal distance, i.e. a sex difference widely acknowledged to be interactionally significant, is similar in size to the effect of the hijab among women in this experiment. Second, it is obvious that the chance of perceiving an objective change in interpersonal distance depends on the baseline. At more than 3 meters distance, a 5 cm change can hardly go noticed. But a few centimeters apart (for example, at hugging or kissing distance), the same objective change can hardly go unnoticed. Third, consideration of the chance of perceiving a change in interpersonal distance cannot make abstraction of what the modification might be accomplishing. The estimated mean among women lies between 84 and 86 cm. At this particular distance, a 5 cm change might make the difference between being within touching distance and being out of reach. It is not unthinkable that, in the context of an ongoing exchange with a stranger, the fact of being in one zone or the other matters to the interaction partners.

### Alternative interpretations

The counterintuitive finding that passengers’ most common response to the hijab is to show increased involvement by decreasing distance might be contested on the ground that staying closer does not necessarily indicate an increase in involvement in the context under study. More specifically, it could be argued that passengers exhibit shorter distances not because they prefer to stay closer but simply because they fail to move in response to the confederate’s increase in interactional involvement. Two explanations may be invoked to account for passengers’ possible failure to move. On the one hand, passengers might be unwilling to give away what they consider to be their inalienable personal territory. On the other hand, passengers might respond to the increase in involvement with an emotionally motivated freeze reaction. Not involvement, but self-assertion or fear would be the basic meaning of staying closer.

The post hoc models performed to assess if immobility mediates the effect of the hijab on distance do not allow to rule out these alternative explanations for men in Vienna. Among these, the hijab is close to eliciting a credible increase in the probability of staying immobile. But more importantly, when failure to move is controlled, the effect of the hijab on distance undergoes an important decrease in credibility in Vienna, among men principally, and in a derived manner on the average of men and women. In other words, immobility appears to partially mediate the negative effect of the hijab on distance among men in Vienna. However, failure to move does not mediate the negative effect of the hijab on distance within any of the other groups, discarding the suspicion that closer distances may be indicating something other than involvement.

A similar conclusion arises from the model on the probability of helping when the nonverbal outcomes are included as predictors. In general, the nonverbal outcomes do not predict helping, but when they do, the estimated effects support the involvement hypothesis. Among men in Paris a closer distance before the request for help is made increases the probability that the passenger will give a positive answer to it, and a similar but weaker association is in place in Brussels among men. Similarly, among women and men in Paris a longer speaking time weakly raises the probability of eventually helping the confederate.

But granting that the meaning of decreased distance is greater involvement, why is it that women uniformly across the three surveyed sites convey more involvement to the covered confederate by staying closer to her? We predicted this effect from the alternative “victimization” and “agreeableness” hypotheses. According to the victimization hypothesis, women are more likely to regard hijab-wearing women as victims of male oppression, leading to a caring or protective response consisting in helping the confederate more often and spontaneously expressing higher intimacy through nonverbal involvement behaviors. Now, if victimization and the concomitant caring or protective tendency were the true explanation of increased nonverbal involvement, women should exhibit a greater probability of helping the veiled confederate, and that probability should increase as involvement increases because they both obey the same goal and are to the same extent different components of the same behavioral response. However, although they stand closer to the veiled confederate, women do not appear to help her more often. Additionally, among women closer distances do not predict a greater probability of helping, indicating that helping and involvement are uncorrelated in this subsample. According to the alternative agreeableness hypothesis, in interaction with a negatively viewed other, women are more likely to seek to make the interaction less unpleasant, resulting in the deliberate performance of nonverbal behaviors indicative of greater involvement that are not, however, the spontaneous reflection of an underlying positive affect. The agreeableness hypothesis is less demanding than the victimization hypothesis and does not appear to be challenged by the data.

### Three important findings

The first important finding of this study is that in spite of the general opposition that the hijab encounters in the countries where the experiment was performed, there is no general tendency among passengers to be uncooperative with, or to show nonverbal hostility toward, a covered woman in the context of the helping interactions we have provoked and observed on metro platforms. In actuality, the most common responses are to offer help with the same probability as when the confederate appears with uncovered hair and to show higher, not lower, nonverbal involvement by interacting at closer distances. This finding cannot be explained away by the operation of ingroup favoritism: the intensification of interpersonal involvement that the hijab elicits concerns mainly non-Muslim passengers.

The second important finding is that this counterintuitive tendency to show more, not less, involvement in interaction with a devalued other is more pronounced among women. This tendency is not altogether absent from the groups of male passengers, but these failed to exhibit a trend consistent across sites. Additionally, as suggested by our mediational analyses, it cannot be discarded that the closer distances that the hijab elicits among men in Vienna are actually indicating something other than involvement. Our results thus corroborate those of a previous study in the Paris metro in which women, but not men, interacted at closer distances with a woman when she was recognizable as a Roma, as compared to a control condition in which she was not [28].

The third important finding is that the behavioral manifestation of the generally negative view of the hijab in the site where negativity is known to be greatest, i.e. Paris, is not monolithic hostility. True, only in Paris do passengers tend to offer assistance less often to the covered confederate, and only in Paris do passengers (especially men) indicate decreased involvement by reducing eye contact. But, considering that men in Vienna might not be increasing involvement with their shorter distances, it is also only in Paris that both men and women show greater involvement by staying closer to the hijab-wearing confederate. It is important to emphasize that the nonverbal involvement outcomes are uncorrelated, as indicated by the fact that the results of the model on gaze are indifferent to the inclusion or exclusion of distance as a predictor. What could be at work in Paris, then, is a polarization of the responses elicited by the hijab. On the negative pole, some passengers withhold help and/or decrease involvement with lower eye contact. On the positive pole, other passengers increase involvement by interacting closer. As suggested by the post hoc model on helping, the male passengers that in Paris stay closer to the confederate before the request for help is made have a credibly greater chance of responding cooperatively.

## Conclusion

This three-site field experiment sought to investigate how attitudes to the Islamic headscarf in Europe, as captured by representative surveys, translate into situated behaviors in the context of an interpersonal encounter with an unacquainted hijab-wearing woman in a public place. In particular, we explored the relationship between those attitudes and items of nonverbal behavior that generally indicate interpersonal involvement. One important finding of the present research is that, contrary to intuition, nonverbal behavior, or at least the part of it that bears proxemic import, does not seem to be as automatic and out of conscious control as it has sometimes been portrayed. Non Muslim passengers, especially non Muslim women, exhibited more, not less proxemic involvement when they interacted with the veiled confederate, exaggerating their display of affective engagement compared to the control condition. This, we think, is an unexpected but historically important phenomenon that, if corroborated by future work, ought to be analyzed in its distal and immediate causes as well as in its consequences for social life.

## Supporting information

S1 File Supplementary material, method. (PDF)

S2 File Post hoc analysis ingroup favoritism. (ZIP)

S3 File (ZIP)
