# Correction: Responses to the Islamic headscarf in everyday interactions depend on sex and locale: A field experiment in the metros of Brussels, Paris, and Vienna on helping and involvement behaviors

**DOI:** 10.1371/journal.pone.0302310

**Published:** 2024-04-11

**Authors:** Martin Aranguren, Francesco Madrisotti, Eser Durmaz-Martins, Gernot Gerger, Lena Wittmann, Marc Méhu

In the Funding section, the grant number is missing for the Austrian Science Fund FWF. The grant number is: I 3645-G29.

There is an error in affiliation 2 for authors Gernot Gerger and Lena Wittmann. The correct affiliation 2 is: Department of Psychology, Webster Vienna Private University, Vienna, Austria.
