# Supplementary material for: Responses to the Islamic headscarf in everyday interactions depend on sex and locale: A field experiment in the metros of Brussels, Paris, and Vienna on helping and involvement behaviors
Source: PLoS One. 2021 Jul 29;16(7):e0254927. doi: 10.1371/journal.pone.0254927 (PMC8321112; doi:10.1371/journal.pone.0254927)
Supplement: S1 File — (PDF) [file pone.0254927.s001.pdf]

Supplementary material: Method

Experimental conditions

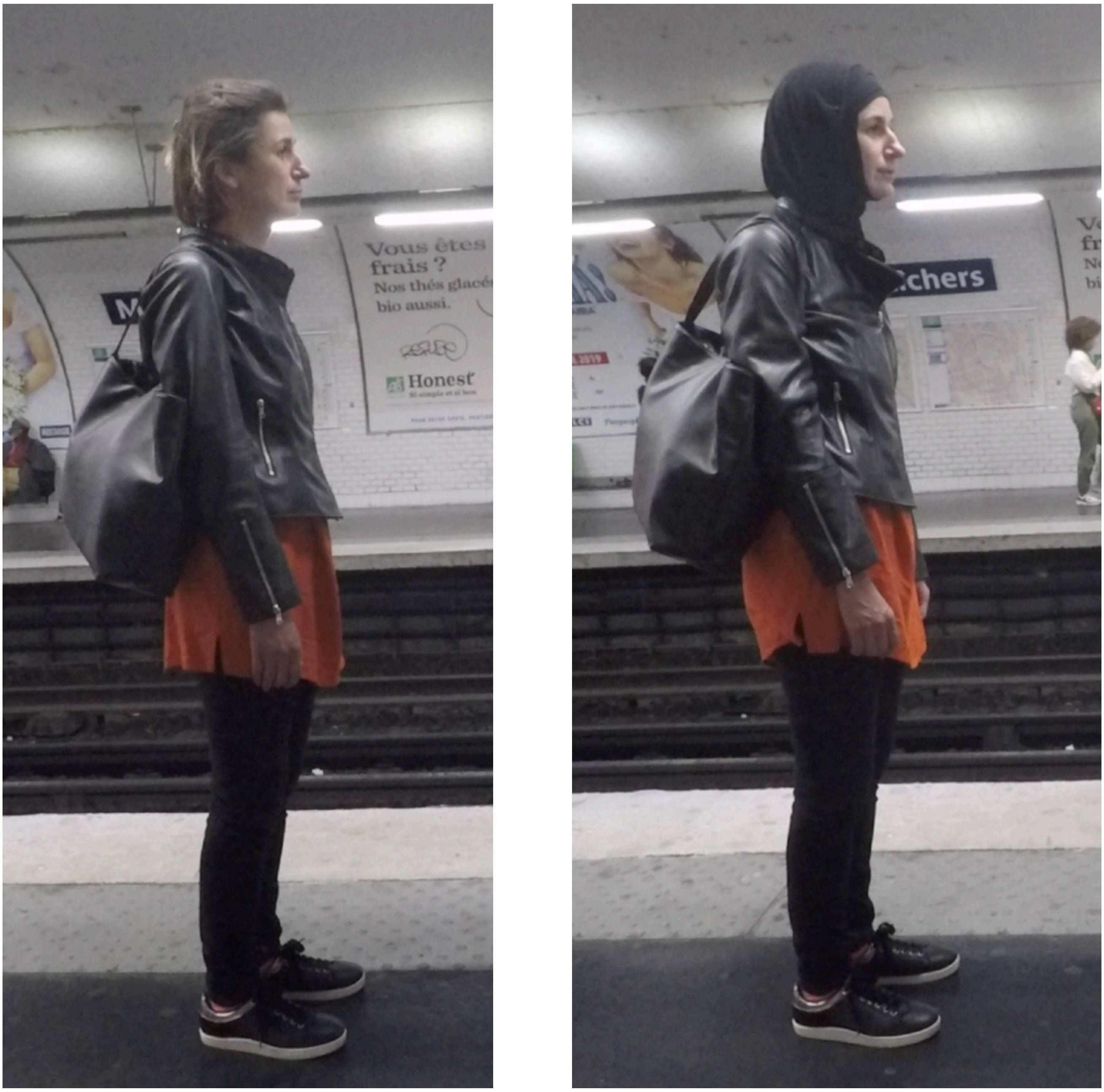

Figure 1: control condition (left); hijab condition (right)

Experimenter 1

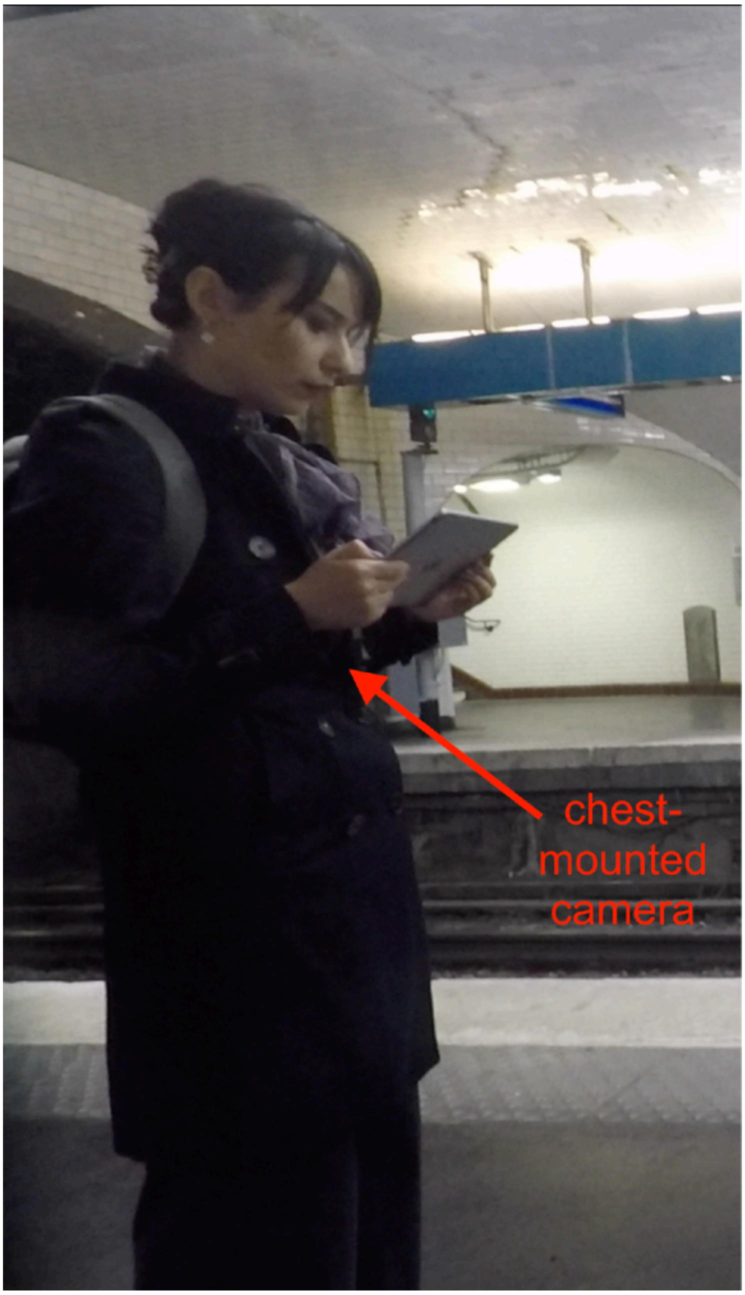

Figure 2: Appearance of Experimenter 1
