# Supplementary material for: Responses to the Islamic headscarf in everyday interactions depend on sex and locale: A field experiment in the metros of Brussels, Paris, and Vienna on helping and involvement behaviors
Source: PLoS One. 2021 Jul 29;16(7):e0254927. doi: 10.1371/journal.pone.0254927 (PMC8321112; doi:10.1371/journal.pone.0254927)
Supplement: S3 File — (ZIP) [file pone.0254927.s003.zip › centralPosteriorIntervalsTables.pdf]

probability of helping, expressed as %

parameter	percentile						
	2.5	5	25	50	75	95	97.5
mainEffect	-12.304	-11.117	-7.566	-5.012	-2.489	1.109	2.257
Bruf	-17.385	-15.134	-8.173	-3.463	1.254	7.971	10.342
Parf	-29.811	-27.183	-19.008	-13.208	-7.449	0.935	3.659
Vief	-6.894	-4.528	2.984	8.122	13.2	20.458	23.018
Brum	-19.277	-16.609	-9.033	-3.809	1.335	8.855	11.402
Parm	-23.36	-20.975	-13.019	-7.361	-1.737	6.532	9.092
Viem	-25.851	-23.161	-14.975	-9.164	-3.304	5.149	7.952
BruParVief	-11.626	-10.264	-5.906	-2.871	0.164	4.529	5.933
BruParViem	-16.051	-14.649	-10	-6.804	-3.534	1.12	2.653
Brufm	-14.034	-12.279	-7.176	-3.647	-0.129	4.916	6.615
Parfm	-21.82	-19.99	-14.34	-10.325	-6.25	-0.368	1.553
Viefm	-11.803	-9.977	-4.44	-0.553	3.402	8.961	10.792
b0 (grand mean)	32.657	33.336	35.308	36.672	38.004	39.957	40.56
b.cond[control]	-1.122	-0.553	1.253	2.543	3.867	5.745	6.367
b.cond[hijab]	-5.935	-5.384	-3.701	-2.471	-1.236	0.556	1.137
b.sex[female]	-8.356	-7.781	-6.01	-4.737	-3.457	-1.519	-0.884
b.sex[male]	0.895	1.547	3.601	5.018	6.455	8.516	9.215
b.site[Bru]	-19.332	-18.72	-16.752	-15.315	-13.839	-11.622	-10.86
b.site[Par]	15.847	16.683	19.331	21.193	23.027	25.677	26.561
b.site[Vie]	-7.291	-6.5	-4.086	-2.36	-0.591	2.019	2.89
b.cond.sex[control, female]	-4.659	-4.09	-2.416	-1.211	0.015	1.836	2.412
b.cond.sex[hijab, female]	-2.128	-1.612	0.032	1.183	2.362	4.064	4.631
b.cond.sex[control, male]	-2.624	-2.036	-0.082	1.27	2.624	4.557	5.197
b.cond.sex[hijab, male]	-4.892	-4.315	-2.535	-1.257	0.037	1.965	2.548
b.cond.site[control, Bru]	-4.581	-3.945	-2.015	-0.616	0.855	3.068	3.819
b.cond.site[hijab, Bru]	-2.813	-2.27	-0.506	0.746	2.029	3.979	4.636
b.cond.site[control, Par]	-2.549	-1.711	0.843	2.567	4.284	6.669	7.455
b.cond.site[hijab, Par]	-8.221	-7.348	-4.713	-2.884	-1.057	1.571	2.384
b.cond.site[control, Vie]	-7.388	-6.6	-4.25	-2.58	-0.899	1.598	2.394
b.cond.site[hijab, Vie]	-2.176	-1.455	0.872	2.521	4.213	6.709	7.599
b.sex.site[female, Bru]	-0.669	-0.08	1.79	3.128	4.551	6.756	7.509
b.sex.site[male, Bru]	-7.362	-6.732	-4.708	-3.208	-1.633	0.87	1.71
b.sex.site[female, Par]	-8.189	-7.293	-4.58	-2.731	-0.866	1.806	2.657
b.sex.site[male, Par]	-3.133	-2.316	0.272	2.025	3.762	6.304	7.062
b.sex.site[female, Vie]	-4.919	-4.152	-1.867	-0.238	1.43	3.79	4.604
b.sex.site[male, Vie]	-4.898	-4.09	-1.534	0.35	2.249	5.025	5.901
b.cond.sex.site[control, female, Bru]	-2.718	-2.047	-0.172	1.127	2.496	4.632	5.334
b.cond.sex.site[hijab, female, Bru]	-4.438	-3.905	-2.199	-0.998	0.178	2.028	2.713
b.cond.sex.site[control, male, Bru]	-5.38	-4.665	-2.611	-1.14	0.349	2.687	3.56
b.cond.sex.site[hijab, male, Bru]	-2.613	-1.992	-0.097	1.217	2.571	4.652	5.401
b.cond.sex.site[control, female, Par]	-2.481	-1.635	0.894	2.661	4.446	6.96	7.782
b.cond.sex.site[hijab, female, Par]	-7.626	-6.792	-4.264	-2.456	-0.681	1.847	2.709
b.cond.sex.site[control, male, Par]	-7.701	-6.909	-4.514	-2.877	-1.268	0.942	1.672
b.cond.sex.site[hijab, male, Par]	-2.344	-1.462	0.977	2.704	4.461	6.895	7.697
b.cond.sex.site[control, female, Vie]	-6.89	-6.153	-4.024	-2.562	-1.089	1.036	1.739
b.cond.sex.site[hijab, female, Vie]	-1.612	-0.892	1.379	2.998	4.627	7.13	7.964
b.cond.sex.site[control, male, Vie]	-1.763	-0.946	1.61	3.428	5.226	7.891	8.797
b.cond.sex.site[hijab, male, Vie]	-8.098	-7.323	-4.92	-3.278	-1.592	0.893	1.689

distance in cm

parameter	percentile						
	2.5	5	25	50	75	95	97.5
mainEffect	4.039	3.751	2.914	2.318	1.729	0.873	0.587
Bruf	7.465	6.765	4.631	3.162	1.722	-0.397	-1.055
Parf	7.884	7.358	5.684	4.563	3.424	1.769	1.211
Vief	6.164	5.54	3.686	2.428	1.174	-0.584	-1.182
Brum	3.542	2.534	-0.439	-2.548	-4.672	-7.723	-8.747
Parm	8.052	7.378	5.264	3.796	2.345	0.184	-0.536
Viem	5.328	4.873	3.5	2.519	1.543	0.121	-0.324
BruParVief	5.547	5.2	4.135	3.395	2.635	1.559	1.226
BruParViem	3.899	3.48	2.177	1.25	0.33	-0.98	-1.409
Brufm	4.028	3.423	1.606	0.313	-0.988	-2.816	-3.443
Parfm	6.864	6.446	5.105	4.174	3.248	1.883	1.445
Viefm	4.813	4.425	3.268	2.475	1.684	0.546	0.177
b0 (grand mean)	-86.263	-86.406	-86.827	-87.126	-87.422	-87.856	-88.001
b.cond[control]	-0.294	-0.436	-0.864	-1.159	-1.457	-1.876	-2.02
b.cond[hijab]	2.02	1.876	1.457	1.159	0.864	0.436	0.294
b.sex[female]	3.023	2.887	2.469	2.168	1.876	1.453	1.31
b.sex[male]	-1.31	-1.453	-1.876	-2.168	-2.469	-2.887	-3.023
b.site[Bru]	2.487	2.255	1.574	1.091	0.614	-0.078	-0.311
b.site[Par]	5.159	4.97	4.387	3.988	3.59	3.018	2.815
b.site[Vie]	-3.985	-4.152	-4.698	-5.083	-5.457	-6.003	-6.194
b.cond.sex[control, female]	0.319	0.183	-0.238	-0.534	-0.83	-1.255	-1.395
b.cond.sex[hijab, female]	1.395	1.255	0.83	0.534	0.238	-0.183	-0.319
b.cond.sex[control, male]	1.395	1.255	0.83	0.534	0.238	-0.183	-0.319
b.cond.sex[hijab, male]	0.319	0.183	-0.238	-0.534	-0.83	-1.255	-1.395
b.cond.site[control, Bru]	2.395	2.169	1.482	1.004	0.531	-0.143	-0.357
b.cond.site[hijab, Bru]	0.357	0.143	-0.531	-1.004	-1.482	-2.169	-2.395
b.cond.site[control, Par]	0.228	0.04	-0.522	-0.929	-1.321	-1.913	-2.106
b.cond.site[hijab, Par]	2.106	1.913	1.321	0.929	0.522	-0.04	-0.228
b.cond.site[control, Vie]	0.993	0.829	0.298	-0.081	-0.452	-0.993	-1.163
b.cond.site[hijab, Vie]	1.163	0.993	0.452	0.081	-0.298	-0.829	-0.993
b.sex.site[female, Bru]	2.249	2.022	1.323	0.848	0.37	-0.311	-0.541
b.sex.site[male, Bru]	0.541	0.311	-0.37	-0.848	-1.323	-2.022	-2.249
b.sex.site[female, Par]	2.149	1.958	1.394	0.993	0.589	0.01	-0.166
b.sex.site[male, Par]	0.166	-0.01	-0.589	-0.993	-1.394	-1.958	-2.149
b.sex.site[female, Vie]	-0.742	-0.921	-1.463	-1.833	-2.217	-2.754	-2.925
b.sex.site[male, Vie]	2.925	2.754	2.217	1.833	1.463	0.921	0.742
b.cond.sex.site[control, female, Bru]	0.473	0.253	-0.427	-0.893	-1.376	-2.061	-2.294
b.cond.sex.site[hijab, female, Bru]	2.294	2.061	1.376	0.893	0.427	-0.253	-0.473
b.cond.sex.site[control, male, Bru]	2.294	2.061	1.376	0.893	0.427	-0.253	-0.473
b.cond.sex.site[hijab, male, Bru]	0.473	0.253	-0.427	-0.893	-1.376	-2.061	-2.294
b.cond.sex.site[control, female, Par]	1.508	1.317	0.746	0.344	-0.058	-0.631	-0.815
b.cond.sex.site[hijab, female, Par]	0.815	0.631	0.058	-0.344	-0.746	-1.317	-1.508
b.cond.sex.site[control, male, Par]	0.815	0.631	0.058	-0.344	-0.746	-1.317	-1.508
b.cond.sex.site[hijab, male, Par]	1.508	1.317	0.746	0.344	-0.058	-0.631	-0.815
b.cond.sex.site[control, female, Vie]	1.648	1.467	0.926	0.553	0.181	-0.363	-0.548
b.cond.sex.site[hijab, female, Vie]	0.548	0.363	-0.181	-0.553	-0.926	-1.467	-1.648
b.cond.sex.site[control, male, Vie]	0.548	0.363	-0.181	-0.553	-0.926	-1.467	-1.648
b.cond.sex.site[hijab, male, Vie]	1.648	1.467	0.926	0.553	0.181	-0.363	-0.548
nu[female, Bru]	-4.871	-5.929	-12.338	-22.821	-40.979	-85.103	-104.958
nu[male, Bru]	-2.021	-2.18	-2.821	-3.391	-4.119	-5.564	-6.144
nu[female, Par]	-3.354	-3.833	-6.669	-11.742	-23.393	-59.639	-76.36
nu[male,Par]	-4.579	-5.235	-8.192	-11.981	-18.816	-40.873	-53.852
nu[female, Vie]	-3.332	-3.87	-6.834	-12.133	-24.385	-61.314	-79.738
nu[male, Vie]	-1.817	-1.967	-2.535	-3.063	-3.71	-5.011	-5.559
sigma.y[female, Bru]	-9.464	-9.808	-10.799	-11.429	-12.058	-12.981	-13.297
sigma.y[male, Bru]	-11.225	-11.618	-12.938	-13.91	-14.919	-16.444	-16.963
sigma.y[female, Par]	-7.216	-7.549	-8.538	-9.222	-9.848	-10.68	-10.953
sigma.y[male, Par]	-9.744	-10.022	-10.888	-11.515	-12.182	-13.179	-13.502
sigma.y[female, Vie]	-7.732	-8.079	-9.175	-9.88	-10.539	-11.445	-11.719
sigma.y[male, Vie]	-5.099	-5.3	-5.968	-6.444	-6.948	-7.717	-8.003

gaze rate, expressed as proportions

parameter	percentile						
	2.5	5	25	50	75	95	97.5
mainEffect	-0.029	-0.025	-0.012	-0.003	0.006	0.018	0.022
Bruf	-0.067	-0.055	-0.016	0.011	0.038	0.077	0.09
Parf	-0.084	-0.073	-0.04	-0.016	0.007	0.041	0.051
Vief	-0.028	-0.022	-0.003	0.011	0.024	0.044	0.05
Brum	-0.082	-0.07	-0.035	-0.01	0.015	0.05	0.062
Parm	-0.137	-0.126	-0.094	-0.072	-0.049	-0.016	-0.005
Viem	-0.021	-0.013	0.009	0.024	0.04	0.063	0.07
BruParVief	-0.035	-0.029	-0.011	0.002	0.015	0.032	0.039
BruParViem	-0.055	-0.049	-0.031	-0.019	-0.007	0.011	0.017
Brufm	-0.053	-0.044	-0.018	0.001	0.019	0.045	0.054
Parfm	-0.091	-0.083	-0.06	-0.044	-0.028	-0.004	0.003
Viefm	-0.012	-0.007	0.007	0.017	0.028	0.043	0.048
b0 (grand mean)	0.749	0.752	0.76	0.765	0.77	0.778	0.78
b.cond[control]	-0.011	-0.009	-0.003	0.002	0.006	0.012	0.014
b.cond[hijab]	-0.015	-0.013	-0.006	-0.002	0.003	0.009	0.011
b.sex[female]	-0.009	-0.006	0.001	0.007	0.012	0.019	0.021
b.sex[male]	-0.023	-0.02	-0.012	-0.007	-0.001	0.006	0.009
b.site[Bru]	-0.128	-0.124	-0.112	-0.103	-0.095	-0.083	-0.08
b.site[Par]	-0.068	-0.065	-0.053	-0.046	-0.038	-0.028	-0.024
b.site[Vie]	0.093	0.096	0.103	0.108	0.113	0.119	0.122
b.cond.sex[control, female]	-0.016	-0.014	-0.008	-0.004	0.001	0.007	0.009
b.cond.sex[hijab, female]	-0.009	-0.007	-0.001	0.004	0.008	0.014	0.016
b.cond.sex[control, male]	-0.009	-0.007	-0.001	0.004	0.008	0.014	0.016
b.cond.sex[hijab, male]	-0.017	-0.015	-0.008	-0.004	0.001	0.007	0.009
b.cond.site[control, Bru]	-0.024	-0.021	-0.01	-0.002	0.006	0.017	0.02
b.cond.site[hijab, Bru]	-0.021	-0.017	-0.006	0.002	0.01	0.02	0.024
b.cond.site[control, Par]	0	0.003	0.013	0.019	0.026	0.035	0.038
b.cond.site[hijab, Par]	-0.041	-0.038	-0.028	-0.02	-0.013	-0.003	0
b.cond.site[control, Vie]	-0.024	-0.021	-0.015	-0.011	-0.006	0	0.002
b.cond.site[hijab, Vie]	-0.002	0	0.006	0.01	0.014	0.02	0.022
b.sex.site[female, Bru]	-0.036	-0.032	-0.02	-0.012	-0.004	0.008	0.012
b.sex.site[male, Bru]	-0.012	-0.008	0.004	0.012	0.02	0.032	0.036
b.sex.site[female, Par]	-0.012	-0.009	0.002	0.01	0.017	0.027	0.03
b.sex.site[male, Par]	-0.033	-0.029	-0.018	-0.01	-0.002	0.009	0.012
b.sex.site[female, Vie]	-0.015	-0.013	-0.006	-0.001	0.004	0.01	0.012
b.sex.site[male, Vie]	-0.015	-0.012	-0.004	0.001	0.006	0.012	0.015
b.cond.sex.site[control, female, Bru]	-0.025	-0.021	-0.009	-0.002	0.006	0.017	0.02
b.cond.sex.site[hijab, female, Bru]	-0.021	-0.017	-0.006	0.002	0.009	0.02	0.024
b.cond.sex.site[control, male, Bru]	-0.021	-0.017	-0.006	0.001	0.009	0.02	0.023
b.cond.sex.site[hijab, male, Bru]	-0.024	-0.02	-0.009	-0.001	0.006	0.017	0.02
b.cond.sex.site[control, female, Par]	-0.029	-0.026	-0.016	-0.009	-0.003	0.006	0.009
b.cond.sex.site[hijab, female, Par]	-0.01	-0.007	0.003	0.01	0.017	0.026	0.029
b.cond.sex.site[control, male, Par]	-0.01	-0.007	0.003	0.01	0.016	0.026	0.029
b.cond.sex.site[hijab, male, Par]	-0.034	-0.03	-0.019	-0.011	-0.004	0.007	0.01
b.cond.sex.site[control, female, Vie]	-0.006	-0.003	0.003	0.007	0.011	0.017	0.019
b.cond.sex.site[hijab, female, Vie]	-0.018	-0.017	-0.011	-0.007	-0.003	0.003	0.005
b.cond.sex.site[control, male, Vie]	-0.022	-0.019	-0.012	-0.007	-0.003	0.003	0.006
b.cond.sex.site[hijab, male, Vie]	-0.005	-0.003	0.003	0.007	0.011	0.017	0.019
kappa[female, Bru]	2.552	2.652	2.974	3.221	3.477	3.865	3.993
kappa[male, Bru]	3.403	3.543	3.998	4.343	4.703	5.268	5.461
kappa[female, Par]	2.339	2.426	2.714	2.929	3.15	3.489	3.603
kappa[male,Par]	2.91	3.025	3.381	3.644	3.933	4.36	4.509
kappa[female, Vie]	1.915	2.004	2.313	2.549	2.806	3.205	3.342
kappa[male, Vie]	2.118	2.219	2.567	2.829	3.115	3.553	3.708

speech rate, expressed as proportions

parameter	percentile						
	2.5	5	25	50	75	95	97.5
mainEffect	-0.021	-0.018	-0.009	-0.003	0.004	0.013	0.016
Bruf	-0.063	-0.056	-0.033	-0.017	-0.002	0.021	0.028
Parf	-0.087	-0.08	-0.054	-0.037	-0.02	0.005	0.013
Vief	-0.046	-0.038	-0.017	-0.002	0.013	0.035	0.042
Brum	-0.02	-0.014	0.007	0.022	0.037	0.058	0.065
Parm	-0.038	-0.031	-0.01	0.005	0.019	0.04	0.047
Viem	-0.034	-0.027	-0.005	0.01	0.025	0.046	0.053
BruParVief	-0.046	-0.041	-0.028	-0.019	-0.01	0.004	0.008
BruParViem	-0.013	-0.009	0.004	0.012	0.021	0.033	0.037
Brufm	-0.029	-0.024	-0.009	0.002	0.013	0.029	0.033
Parfm	-0.049	-0.044	-0.028	-0.016	-0.005	0.011	0.017
Viefm	-0.027	-0.022	-0.006	0.004	0.015	0.03	0.035
b0 (grand mean)	0.3	0.302	0.306	0.309	0.313	0.317	0.319
b.cond[control]	-0.008	-0.006	-0.002	0.001	0.005	0.009	0.011
b.cond[hijab]	-0.011	-0.009	-0.005	-0.001	0.002	0.006	0.008
b.sex[female]	0.008	0.009	0.014	0.017	0.02	0.025	0.027
b.sex[male]	-0.025	-0.024	-0.02	-0.017	-0.014	-0.009	-0.008
b.site[Bru]	0.021	0.023	0.03	0.034	0.039	0.045	0.048
b.site[Par]	-0.005	-0.003	0.003	0.008	0.013	0.019	0.021
b.site[Vie]	-0.052	-0.05	-0.044	-0.04	-0.036	-0.029	-0.027
b.cond.sex[control, female]	-0.002	0	0.005	0.008	0.011	0.016	0.018
b.cond.sex[hijab, female]	-0.017	-0.016	-0.011	-0.008	-0.004	0	0.002
b.cond.sex[control, male]	-0.016	-0.015	-0.01	-0.007	-0.004	0	0.002
b.cond.sex[hijab, male]	-0.002	0	0.004	0.007	0.011	0.015	0.017
b.cond.site[control, Bru]	-0.016	-0.014	-0.007	-0.003	0.002	0.009	0.011
b.cond.site[hijab, Bru]	-0.01	-0.008	-0.002	0.003	0.007	0.014	0.016
b.cond.site[control, Par]	-0.007	-0.005	0.002	0.006	0.011	0.018	0.02
b.cond.site[hijab, Par]	-0.019	-0.017	-0.011	-0.006	-0.002	0.005	0.007
b.cond.site[control, Vie]	-0.016	-0.014	-0.008	-0.004	0.001	0.007	0.009
b.cond.site[hijab, Vie]	-0.009	-0.007	-0.001	0.004	0.008	0.014	0.016
b.sex.site[female, Bru]	-0.024	-0.022	-0.016	-0.011	-0.007	0	0.002
b.sex.site[male, Bru]	-0.002	0	0.006	0.011	0.015	0.022	0.024
b.sex.site[female, Par]	-0.006	-0.004	0.003	0.008	0.012	0.019	0.022
b.sex.site[male, Par]	-0.02	-0.018	-0.011	-0.007	-0.003	0.004	0.006
b.sex.site[female, Vie]	-0.01	-0.008	-0.002	0.003	0.007	0.014	0.016
b.sex.site[male, Vie]	-0.014	-0.012	-0.007	-0.003	0.002	0.008	0.01
b.cond.sex.site[control, female, Bru]	-0.011	-0.009	-0.003	0.002	0.007	0.013	0.016
b.cond.sex.site[hijab, female, Bru]	-0.015	-0.013	-0.007	-0.002	0.002	0.009	0.011
b.cond.sex.site[control, male, Bru]	-0.015	-0.013	-0.007	-0.002	0.002	0.008	0.011
b.cond.sex.site[hijab, male, Bru]	-0.011	-0.009	-0.002	0.002	0.007	0.014	0.016
b.cond.sex.site[control, female, Par]	-0.011	-0.009	-0.002	0.003	0.008	0.015	0.018
b.cond.sex.site[hijab, female, Par]	-0.016	-0.014	-0.007	-0.003	0.002	0.009	0.011
b.cond.sex.site[control, male, Par]	-0.016	-0.014	-0.007	-0.003	0.002	0.008	0.01
b.cond.sex.site[hijab, male, Par]	-0.01	-0.008	-0.002	0.003	0.007	0.014	0.016
b.cond.sex.site[control, female, Vie]	-0.017	-0.015	-0.009	-0.005	0	0.006	0.008
b.cond.sex.site[hijab, female, Vie]	-0.008	-0.006	0	0.005	0.009	0.015	0.018
b.cond.sex.site[control, male, Vie]	-0.007	-0.005	0.001	0.005	0.009	0.014	0.016
b.cond.sex.site[hijab, male, Vie]	-0.016	-0.014	-0.009	-0.005	0	0.006	0.008
kappa[female, Bru]	9.761	10.185	11.544	12.547	13.621	15.292	15.856
kappa[male, Bru]	11.582	12.109	13.783	15.035	16.386	18.446	19.136
kappa[female, Par]	7.649	7.937	8.908	9.629	10.395	11.588	12.01
kappa[male,Par]	9.008	9.392	10.582	11.472	12.386	13.797	14.241
kappa[female, Vie]	8.358	8.667	9.735	10.531	11.393	12.692	13.12
kappa[male, Vie]	8.51	8.852	10.032	10.896	11.815	13.246	13.765

probability of making no steps, expressed as %

parameter	percentile						
	2.5	5	25	50	75	95	97.5
mainEffect	-3.075	-2.228	0.468	2.357	4.232	6.942	7.825
Bruf	-17.17	-14.964	-8.439	-4.042	0.28	6.751	8.888
Parf	-7.404	-6.187	-2.615	-0.39	1.754	5.273	6.547
Vief	-18.776	-16.184	-8.106	-2.524	3.191	11.366	13.96
Brum	-4.801	-3.116	1.617	4.792	8.242	13.69	15.702
Parm	-6.26	-4.652	-0.102	2.782	5.726	10.487	12.054
Viem	-2.937	-0.354	7.848	13.458	18.961	26.977	29.53
BruParVief	-9.706	-8.472	-4.84	-2.314	0.197	3.838	5.004
BruParViem	0.019	1.153	4.61	7.046	9.498	13.043	14.141
Brufm	-7.755	-6.371	-2.385	0.416	3.267	7.414	8.798
Parfm	-4.549	-3.544	-0.71	1.198	3.102	5.995	6.996
Viefm	-6.164	-4.168	1.506	5.458	9.419	15.114	16.961
b0 (grand mean)	11.59	12.043	13.403	14.384	15.428	16.942	17.484
b.cond[control]	-3.479	-3.124	-1.984	-1.139	-0.233	1.15	1.602
b.cond[hijab]	-1.468	-1.077	0.235	1.22	2.244	3.815	4.348
b.sex[female]	-1.91	-1.442	-0.034	1.005	2.111	3.784	4.363
b.sex[male]	-3.483	-3.102	-1.88	-0.949	0.034	1.572	2.145
b.site[Bru]	-5.571	-5.106	-3.531	-2.344	-1.057	0.951	1.659
b.site[Par]	-10.169	-9.832	-8.79	-8.035	-7.246	-6.042	-5.622
b.site[Vie]	14.323	15.193	17.847	19.744	21.7	24.542	25.434
b.cond.sex[control, female]	-0.539	-0.122	1.212	2.227	3.332	5.041	5.685
b.cond.sex[hijab, female]	-4.882	-4.461	-3.158	-2.233	-1.307	0.124	0.633
b.cond.sex[control, male]	-3.932	-3.559	-2.446	-1.689	-0.947	0.138	0.514
b.cond.sex[hijab, male]	-0.564	-0.135	1.247	2.267	3.367	5.07	5.704
b.cond.site[control, Bru]	-2.612	-2.125	-0.694	0.334	1.422	3.202	3.827
b.cond.site[hijab, Bru]	-3.586	-3.076	-1.484	-0.334	0.921	2.905	3.588
b.cond.site[control, Par]	-1.171	-0.845	0.141	0.82	1.515	2.614	3.008
b.cond.site[hijab, Par]	-3.044	-2.669	-1.509	-0.732	0.048	1.235	1.67
b.cond.site[control, Vie]	-6.881	-6.082	-3.673	-1.896	-0.104	2.601	3.543
b.cond.site[hijab, Vie]	-3.652	-2.772	-0.045	1.867	3.853	6.716	7.731
b.sex.site[female, Bru]	-0.638	-0.007	1.939	3.438	5.026	7.594	8.472
b.sex.site[male, Bru]	-5.259	-4.767	-3.295	-2.329	-1.355	0.078	0.61
b.sex.site[female, Par]	-5.179	-4.74	-3.463	-2.631	-1.84	-0.745	-0.422
b.sex.site[male, Par]	0.876	1.236	2.39	3.289	4.317	6.094	6.759
b.sex.site[female, Vie]	-1.767	-0.9	1.876	3.821	5.814	8.776	9.722
b.sex.site[male, Vie]	-8.515	-7.753	-5.351	-3.658	-1.914	0.717	1.577
b.cond.sex.site[control, female, Bru]	-3.545	-2.91	-0.901	0.58	2.253	5.042	6.031
b.cond.sex.site[hijab, female, Bru]	-4.339	-3.728	-1.817	-0.537	0.739	2.777	3.537
b.cond.sex.site[control, male, Bru]	-2.209	-1.722	-0.351	0.514	1.358	2.575	2.959
b.cond.sex.site[hijab, male, Bru]	-3.519	-3.007	-1.422	-0.36	0.789	2.712	3.455
b.cond.sex.site[control, female, Par]	-4.098	-3.659	-2.378	-1.551	-0.735	0.379	0.733
b.cond.sex.site[hijab, female, Par]	-0.611	-0.219	0.963	1.739	2.488	3.596	3.949
b.cond.sex.site[control, male, Par]	-1.35	-0.978	0.077	0.794	1.566	3.036	3.657
b.cond.sex.site[hijab, male, Par]	-4.11	-3.643	-2.189	-1.207	-0.154	1.628	2.378
b.cond.sex.site[control, female, Vie]	-3.747	-2.832	-0.031	1.929	3.915	6.818	7.762
b.cond.sex.site[hijab, female, Vie]	-7.629	-6.771	-4.073	-2.176	-0.227	2.63	3.518
b.cond.sex.site[control, male, Vie]	-5.888	-5.137	-2.964	-1.485	0.012	2.234	3.041
b.cond.sex.site[hijab, male, Vie]	-3.673	-2.829	-0.167	1.71	3.64	6.473	7.426

distance in cm (failure to move controlled)

parameter	percentile						
	2.5	5	25	50	75	95	97.5
mainEffect	3.55	3.3	2.49	1.94	1.38	0.59	0.32
Bruf	7.33	6.66	4.63	3.26	1.88	-0.15	-0.75
Parf	7.75	7.25	5.7	4.62	3.56	2	1.45
Vief	6.2	5.61	3.82	2.61	1.4	-0.36	-0.9
Brum	2.24	1.25	-1.62	-3.59	-5.54	-8.4	-9.32
Parm	7.33	6.73	4.78	3.48	2.16	0.27	-0.35
Viem	3.94	3.48	2.16	1.23	0.3	-1.07	-1.51
BruParVief	5.56	5.22	4.21	3.5	2.79	1.75	1.4
BruParViem	2.85	2.46	1.23	0.38	-0.47	-1.71	-2.11
Brufm	3.35	2.8	1.04	-0.16	-1.37	-3.1	-3.66
Parfm	6.52	6.12	4.91	4.04	3.19	1.96	1.57
Viefm	4.18	3.82	2.68	1.91	1.15	0.05	-0.3
b0 (grand mean)	-82.83	-83	-83.54	-83.92	-84.3	-84.84	-85.03
b.cond[control]	-0.16	-0.3	-0.69	-0.97	-1.24	-1.65	-1.78
b.cond[hijab]	1.78	1.65	1.24	0.97	0.69	0.3	0.16
b.sex[female]	2.89	2.72	2.17	1.79	1.41	0.86	0.68
b.sex[male]	-0.68	-0.86	-1.41	-1.79	-2.17	-2.72	-2.89
b.site[Bru]	3.47	3.2	2.35	1.77	1.17	0.33	0.05
b.site[Par]	7.45	7.17	6.37	5.81	5.25	4.42	4.15
b.site[Vie]	-6.29	-6.49	-7.13	-7.57	-8.02	-8.66	-8.87
b.cond.sex[control, female]	0.02	-0.11	-0.5	-0.78	-1.05	-1.46	-1.59
b.cond.sex[hijab, female]	1.59	1.46	1.05	0.78	0.5	0.11	-0.02
b.cond.sex[control, male]	1.59	1.46	1.05	0.78	0.5	0.11	-0.02
b.cond.sex[hijab, male]	0.02	-0.11	-0.5	-0.78	-1.05	-1.46	-1.59
b.cond.site[control, Bru]	2.34	2.14	1.49	1.05	0.6	-0.04	-0.24
b.cond.site[hijab, Bru]	0.24	0.04	-0.6	-1.05	-1.49	-2.14	-2.34
b.cond.site[control, Par]	0.02	-0.16	-0.69	-1.06	-1.42	-1.96	-2.12
b.cond.site[hijab, Par]	2.12	1.96	1.42	1.06	0.69	0.16	-0.02
b.cond.site[control, Vie]	1.03	0.87	0.36	0.01	-0.35	-0.86	-1.02
b.cond.site[hijab, Vie]	1.02	0.86	0.35	-0.01	-0.36	-0.87	-1.03
b.sex.site[female, Bru]	2.2	1.91	1.03	0.45	-0.14	-0.99	-1.25
b.sex.site[male, Bru]	1.25	0.99	0.14	-0.45	-1.03	-1.91	-2.2
b.sex.site[female, Par]	2.88	2.61	1.8	1.24	0.67	-0.15	-0.41
b.sex.site[male, Par]	0.41	0.15	-0.67	-1.24	-1.8	-2.61	-2.88
b.sex.site[female, Vie]	-0.4	-0.6	-1.23	-1.68	-2.13	-2.76	-2.98
b.sex.site[male, Vie]	2.98	2.76	2.13	1.68	1.23	0.6	0.4
b.cond.sex.site[control, female, Bru]	0.38	0.18	-0.49	-0.93	-1.37	-2.01	-2.21
b.cond.sex.site[hijab, female, Bru]	2.21	2.01	1.37	0.93	0.49	-0.18	-0.38
b.cond.sex.site[control, male, Bru]	2.21	2.01	1.37	0.93	0.49	-0.18	-0.38
b.cond.sex.site[hijab, male, Bru]	0.38	0.18	-0.49	-0.93	-1.37	-2.01	-2.21
b.cond.sex.site[control, female, Par]	1.57	1.4	0.87	0.49	0.12	-0.41	-0.57
b.cond.sex.site[hijab, female, Par]	0.57	0.41	-0.12	-0.49	-0.87	-1.4	-1.57
b.cond.sex.site[control, male, Par]	0.57	0.41	-0.12	-0.49	-0.87	-1.4	-1.57
b.cond.sex.site[hijab, male, Par]	1.57	1.4	0.87	0.49	0.12	-0.41	-0.57
b.cond.sex.site[control, female, Vie]	1.47	1.3	0.79	0.43	0.08	-0.44	-0.6
b.cond.sex.site[hijab, female, Vie]	0.6	0.44	-0.08	-0.43	-0.79	-1.3	-1.47
b.cond.sex.site[control, male, Vie]	0.6	0.44	-0.08	-0.43	-0.79	-1.3	-1.47
b.cond.sex.site[hijab, male, Vie]	1.47	1.3	0.79	0.43	0.08	-0.44	-0.6
nu[female, Bru]	-3.91	-4.66	-9.28	-17.21	-32.41	-74.67	-92.91
nu[male, Bru]	-1.84	-1.97	-2.52	-3.03	-3.68	-4.94	-5.47
nu[female, Par]	-2.68	-3.04	-4.72	-7.28	-13.53	-39.72	-53.55
nu[male,Par]	-3.05	-3.4	-4.91	-6.65	-9.44	-18.16	-23.88
nu[female, Vie]	-3.4	-3.89	-6.83	-11.74	-23.26	-60.47	-78.47
nu[male, Vie]	-1.75	-1.9	-2.45	-2.92	-3.54	-4.78	-5.34
sigma.y[female, Bru]	-8.42	-8.81	-9.91	-10.59	-11.24	-12.14	-12.46
sigma.y[male, Bru]	-10.2	-10.58	-11.89	-12.82	-13.83	-15.38	-15.95
sigma.y[female, Par]	-6.43	-6.71	-7.67	-8.41	-9.11	-10.04	-10.31
sigma.y[male, Par]	-8.09	-8.39	-9.3	-9.96	-10.64	-11.67	-12.03
sigma.y[female, Vie]	-7.6	-7.92	-8.94	-9.64	-10.28	-11.18	-11.47
sigma.y[male, Vie]	-4.73	-4.94	-5.57	-6.02	-6.51	-7.26	-7.51

probability of helping, expressed as %

parameter	percentile						
	2.5	5	25	50	75	95	97.5
mainEffect	-12.43	-11.31	-7.51	-4.89	-2.27	1.6	2.76
Bruf	-17.13	-14.78	-7.67	-2.98	1.72	8.7	10.94
Parf	-28.6	-25.99	-17.22	-11.03	-4.79	4	7.04
Vief	-7.63	-5.21	2.17	7.33	12.35	19.67	22.03
Brum	-16.68	-13.91	-6.47	-1.52	3.39	10.91	13.36
Parm	-29.42	-26.71	-18.53	-12.76	-6.75	1.75	4.58
Viem	-25.37	-22.42	-13.72	-7.51	-1.43	7.61	10.56
BruParVief	-11.26	-9.81	-5.36	-2.24	0.83	5.38	6.79
BruParViem	-16.75	-15.25	-10.55	-7.25	-3.92	0.82	2.37
Brufm	-12.44	-10.75	-5.75	-2.3	1.21	6.28	8.02
Parfm	-24.01	-22.04	-16.11	-11.83	-7.54	-1.46	0.52
Viefm	-11.71	-9.84	-4.17	-0.14	3.9	9.76	11.53
b0 (grand mean)	31.87	32.53	34.55	35.95	37.39	39.4	40.06
b.cond[control]	-1.37	-0.8	1.14	2.48	3.84	5.85	6.44
b.cond[hijab]	-5.99	-5.47	-3.67	-2.41	-1.13	0.8	1.39
b.sex[female]	-8.39	-7.84	-6	-4.68	-3.34	-1.37	-0.75
b.sex[male]	0.75	1.39	3.49	4.96	6.46	8.63	9.29
b.site[Bru]	-20.21	-19.6	-17.64	-16.23	-14.74	-12.5	-11.73
b.site[Par]	17.48	18.37	21.16	23.09	25.03	27.84	28.73
b.site[Vie]	-7.71	-6.93	-4.39	-2.59	-0.79	1.96	2.81
b.cond.sex[control, female]	-4.98	-4.43	-2.69	-1.46	-0.19	1.63	2.24
b.cond.sex[hijab, female]	-1.98	-1.43	0.22	1.42	2.63	4.41	4.97
b.cond.sex[control, male]	-2.45	-1.85	0.15	1.55	2.96	5	5.62
b.cond.sex[hijab, male]	-5.28	-4.68	-2.84	-1.52	-0.18	1.76	2.37
b.cond.site[control, Bru]	-5.17	-4.52	-2.61	-1.24	0.15	2.33	3.09
b.cond.site[hijab, Bru]	-2.32	-1.73	0.08	1.32	2.63	4.62	5.34
b.cond.site[control, Par]	-1.8	-0.97	1.68	3.5	5.24	7.75	8.5
b.cond.site[hijab, Par]	-9.56	-8.67	-5.86	-3.94	-1.99	0.77	1.65
b.cond.site[control, Vie]	-7.58	-6.86	-4.45	-2.72	-0.96	1.6	2.46
b.cond.site[hijab, Vie]	-2.23	-1.46	0.93	2.66	4.44	7	7.89
b.sex.site[female, Bru]	-0.03	0.59	2.45	3.82	5.25	7.49	8.28
b.sex.site[male, Bru]	-7.97	-7.34	-5.4	-3.94	-2.4	-0.01	0.86
b.sex.site[female, Par]	-8.87	-7.92	-5.04	-3.09	-1.12	1.71	2.56
b.sex.site[male, Par]	-3.07	-2.23	0.46	2.29	4.07	6.65	7.51
b.sex.site[female, Vie]	-5.55	-4.79	-2.4	-0.76	0.91	3.39	4.21
b.sex.site[male, Vie]	-4.55	-3.67	-0.94	0.98	2.95	5.87	6.82
b.cond.sex.site[control, female, Bru]	-1.99	-1.38	0.54	1.86	3.24	5.43	6.2
b.cond.sex.site[hijab, female, Bru]	-5.25	-4.68	-2.89	-1.68	-0.45	1.47	2.18
b.cond.sex.site[control, male, Bru]	-5.98	-5.34	-3.3	-1.89	-0.45	1.78	2.59
b.cond.sex.site[hijab, male, Bru]	-1.99	-1.32	0.61	1.93	3.31	5.43	6.25
b.cond.sex.site[control, female, Par]	-4.56	-3.64	-0.92	0.96	2.84	5.49	6.37
b.cond.sex.site[hijab, female, Par]	-6.13	-5.26	-2.55	-0.65	1.24	3.97	4.88
b.cond.sex.site[control, male, Par]	-6.41	-5.62	-3.23	-1.61	0.02	2.35	3.11
b.cond.sex.site[hijab, male, Par]	-4.39	-3.5	-0.78	1.07	2.92	5.52	6.39
b.cond.sex.site[control, female, Vie]	-6.17	-5.46	-3.21	-1.7	-0.22	1.96	2.72
b.cond.sex.site[hijab, female, Vie]	-2.58	-1.82	0.49	2.12	3.83	6.34	7.18
b.cond.sex.site[control, male, Vie]	-2.91	-2.02	0.65	2.51	4.43	7.22	8.11
b.cond.sex.site[hijab, male, Vie]	-7.49	-6.71	-4.2	-2.44	-0.68	1.93	2.8
distance, Bruf	-11.16	-9.65	-4.93	-1.59	1.62	6.02	7.37
distance, Brum	-12.76	-11.53	-7.88	-5.49	-3.26	-0.34	0.54
distance, Parf	-5.94	-4.59	-0.47	2.45	5.23	9.16	10.49
distance, Parm	-18.25	-16.79	-12.31	-9.24	-6.26	-2.33	-1.08
distance, Vief	-16.8	-15.12	-10.08	-6.72	-3.44	0.98	2.46
distance, Viem	-7.71	-6.38	-2.46	0.27	2.97	6.83	8.15
eyeContact, Bruf	-6.84	-5.98	-3.41	-1.6	0.2	2.8	3.61
eyeContact, Brum	-9.23	-8.24	-5.27	-3.26	-1.29	1.43	2.3
eyeContact, Parf	-5.24	-4.52	-2.25	-0.69	0.8	2.94	3.62
eyeContact, Parm	-8.69	-7.79	-5.14	-3.37	-1.64	0.74	1.48
eyeContact, Vief	-6.18	-5.27	-2.59	-0.73	1.16	4	4.99
eyeContact, Viem	-7.96	-7.03	-4.32	-2.47	-0.67	1.92	2.75
speechRate, Bruf	-4.36	-2.86	1.52	4.57	7.55	11.65	12.9
speechRate, Brum	-10.71	-9.02	-3.73	-0.17	3.22	8.12	9.6
speechRate, Parf	-0.56	0.53	3.67	5.81	7.94	11.03	12.02
speechRate, Parm	-0.12	1.14	4.7	7.19	9.64	13.12	14.32
speechRate, Vief	-6.89	-5.57	-1.85	0.61	3.08	6.56	7.66
speechRate, Viem	-7.49	-6.34	-2.6	-0.09	2.33	5.71	6.82
